# Cell-type specific potent Wnt signaling blockade by bispecific antibody

**DOI:** 10.1038/s41598-017-17539-z

**Published:** 2018-01-15

**Authors:** Nam-Kyung Lee, Yafeng Zhang, Yang Su, Scott Bidlingmaier, Daniel W. Sherbenou, Kevin D. Ha, Bin Liu

**Affiliations:** Department of Anesthesia, UCSF Helen Diller Family Comprehensive Cancer Center, 1001 Potrero Ave., 1305, San Francisco, CA 94110-1305 USA

## Abstract

Cell signaling pathways are often shared between normal and diseased cells. How to achieve cell type-specific, potent inhibition of signaling pathways is a major challenge with implications for therapeutic development. Using the Wnt/β-catenin signaling pathway as a model system, we report here a novel and generally applicable method to achieve cell type-selective signaling blockade. We constructed a bispecific antibody targeting the Wnt co-receptor LRP6 (the effector antigen) and a cell type-associated antigen (the guide antigen) that provides the targeting specificity. We found that the bispecific antibody inhibits Wnt-induced reporter activities with over one hundred-fold enhancement in potency, and in a cell type-selective manner. Potency enhancement is dependent on the expression level of the guide antigen on the target cell surface and the apparent affinity of the anti-guide antibody. Both internalizing and non-internalizing guide antigens can be used, with internalizing bispecific antibody being able to block signaling by all ligands binding to the target receptor due to its removal from the cell surface. It is thus feasible to develop bispecific-based therapeutic strategies that potently and selectively inhibit signaling pathways in a cell type-selective manner, creating opportunity for therapeutic targeting.

## Introduction

Cell signaling pathways are critical for maintaining homeostasis and regulating cell growth and survival. Normal and disease cells often use overlapping pathways, creating a roadblock to therapeutic targeting. Selective sensitivity of disease cells to pathway inhibition allows certain pathways to be targeted by inhibitors with some demonstrating clinical applicability^1–3^. Nonetheless, toxicity to normal cells restricts the therapeutic window. It remains a fundamental challenge to achieve cell-type selective inhibition of signaling pathways that are commonly utilized by disease and normal cells.

The Wnt/β-catenin signaling plays important roles in embryonic development and disease pathogenesis^4–6^. Aberrant activation of Wnt signaling has been observed in many types of cancer and plays a role in development of cancer stem-like cells^7–12^. In the canonical Wnt/β-catenin signaling cascade, Wnt ligand binding leads to assembly of a co-receptor complex composed of the 7-transmembrane receptor Frizzled (Fzd) and the low-density lipoprotein receptor-related proteins 5 or 6 (LRP5/6), followed by phosphorylation of LRP5/6. The phosphorylated LRP5/6 sequestrates glycogen synthase kinase 3β (GSK-3β)/Axin complex to the plasma membrane to inhibit β-catenin degradation, allowing stabilized β-catenin to translocate into the nucleus, which then binds to the T-cell factor/lymphoid enhancer factor (TCF/LEF) transcription factors, and induces the expression of various Wnt target genes including cyclin D1 and the proto-oncogene c-Myc^13^.

The extracellular region of LRP6 is comprised of four domains, namely E1 to E4, each including a conserved YWTD β-propeller and EGF-like motif^14^. The ligand-binding sites are separately located on the E1-E2 domain for Wnt1, Wnt2, or Wnt9 and E3-E4 domain for Wnt3 or Wnt3a^15,16^. In addition to Wnt ligands, Norrin or R-spondins (RSPO1 to 4) have been shown to upregulate Wnt/β-catenin signaling by preventing turnover of LRP6^14,17,18^. LRP6 has been considered to be a promising target for therapy development against Wnt-dependent cancers^7,19,20^, but it is also expressed on normal cells, raising concerns of low targeting specificity that may restrict the therapeutic window.

Monoclonal antibodies (mAbs) have emerged as a novel and effective cancer therapeutic, due in part to its high specificity and affinity in target binding, as well as ease of chemical and molecular modifications that enable the development of more complex antibody-based therapeutics^21^. Bispecific antibodies (bsAbs) have emerged as a valid and effective therapeutic^22^. For example, the bispecific T-cell engager (BiTE) Blinatumomab is comprised of an anti-CD19 and an anti-CD3 single-chain variable fragment (scFv) for tumor-targeted T cell recruitment and activation. Other examples include IgG-scFv containing anti-epidermal growth factor receptor 3 (ErbB3) scFv fused to the heavy chain C-termini of an anti-insulin-like growth factor 1 receptor (IGF-1R) IgG, and CrossMAb comprising heterodimeric pairs of two heavy and light chains against vascular endothelial growth factor A (VEGF-A) and angiopoietin-2^23–25^. In a majority of those cases, however, the bispecificity is designed to either introduce a new activity (in the case of BiTE, bringing in a cytotoxic effector function) or to block two pathways critical for cell growth and survival without cell type selectivity (in the case of anti-IGF1R/ErbB3 and VEGF-A/angiopoitein-2). We hypothesize that beyond those known applications, bispecific antibodies can be used to achieve cell-type specific inhibition or activation of signaling pathways, addressing a major challenge in targeted therapy development.

We hereby report a generally applicable approach to achieve cell-type selective signaling pathway modulation by bispecific antibody. We used the Wnt/β-catenin pathway as a model system to demonstrate specificity and potency, and studied other variables such as receptor copy number on cell surface and antibody-induced receptor internalization. We generated anti-LRP6 human mAbs and further bsAbs by joining the anti-LRP6 mAb with a guide antibody targeting a tumor-associated antigen, creating a guide/effector bispecific system. To broaden applicability and investigate the impact of receptor copy number per cell on affinity, specificity and functionality of bsAbs, we studied several tumor-associated cell surface antigens. We have previously identified and characterized human antibodies that target the intercellular adhesion molecule 1 (ICAM-1), ephrin type-A receptor 2 (EphA2), and activated leukocyte cell adhesion molecule (ALCAM)^26–29^. These tumor-associated antigens are overexpressed in multiple cancers^30–32^. We show that when expressed at an above threshold level on the tumor cell surface, antibodies targeting these guide antigens serve as a cell-type selector as well as potency enhancer, resulting in potent and selective inhibition of the Wnt/β-catenin signaling in target cells.

## Results

### Identification of anti-LRP6 scFvs

**Figure 1:**
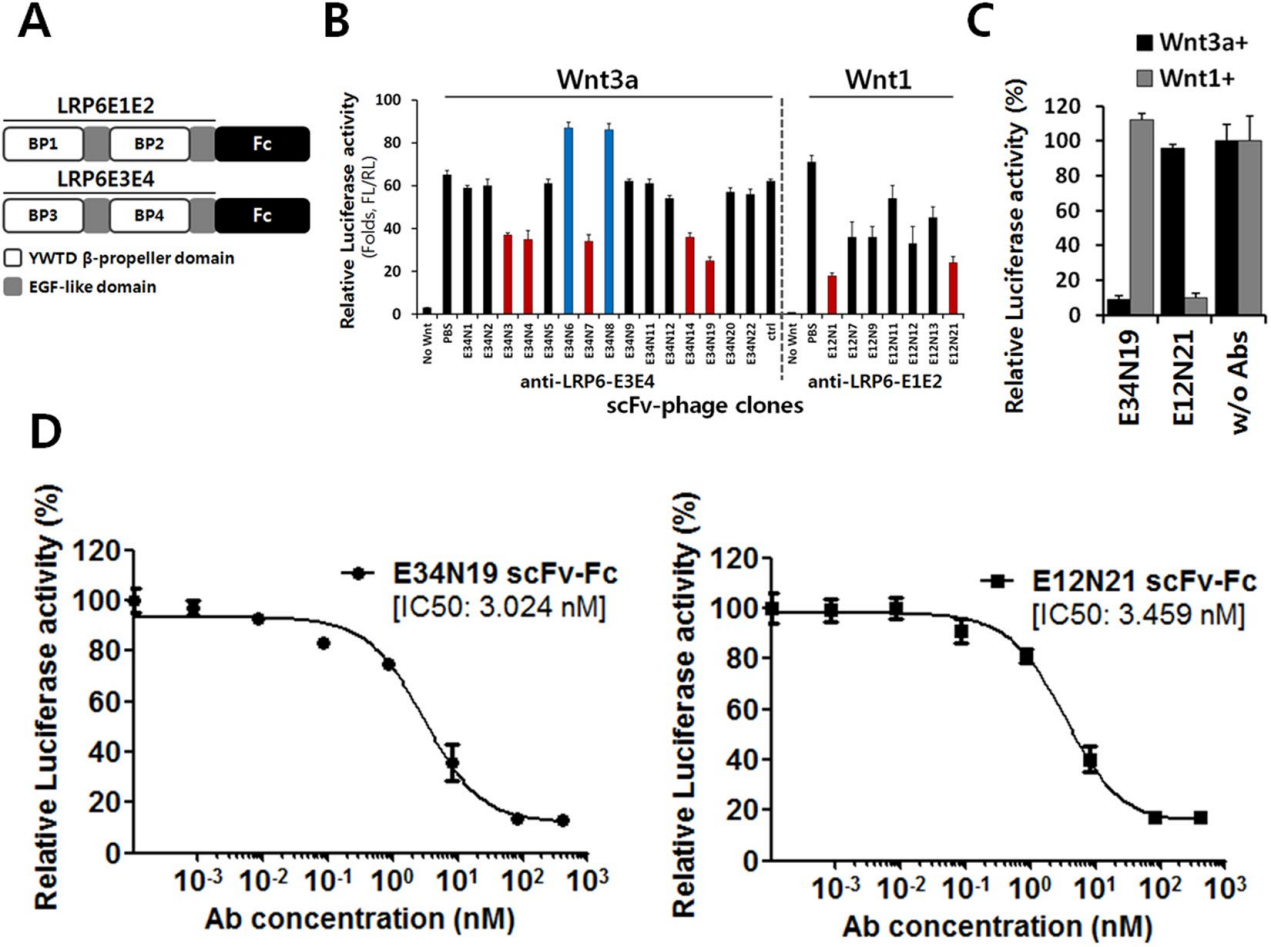
Identification of phage antibodies binding to LRP6 sub-domains. (**a**) A schematic diagram of the extracellular E1E2 and E3E4 domains of LRP6 fused to Fc, each of which contains two β-propeller (BP) domains and two EGF-like domains. Each Fc fusion was purified and used for scFv phage display selection. (**b**) STF reporter assays to screen for functional scFv phage antibodies against LRP6E1E2 or LRP6E3E4 domain. HEK293 cells transfected with STF reporter and Wnt3a- or Wnt1-expression constructs were treated with monoclonal phage antibodies. Relative luciferase activity was calculated by dividing Firefly luciferase (*FL*) reporter activity by co-transfected Renilla Luciferase (*RL*) activity. Both agonistic (*blue*) and antagonistic (*red*) phage antibodies were identified. Error bars represent SD (standard deviation) for n = 3. (**c**) STF reporter assay with scFv-Fc fusion. Purified E34N19 or E12N21 scFv-Fc (100 nM) was tested for Wnt3a- or Wnt1-specific reporter inhibition, respectively. Error bars represent SD for n = 3. (**d**) Potency assessment of β-catenin reporter inhibition by E34N19 and E12N21 scFv-Fc. HEK293 cells were transfected with STF reporter and Wnt3a- or Wnt1-expression constructs and treated with various concentrations of E34N19 or E12N21 scFv-Fc, respectively. IC50 values are indicated on the graph (mean ± SD for n = 3).

To inhibit Wnt/β-catenin signaling induced by Wnt ligand binding to LRP6, we first generated human scFvs binding to the E1E2 and E3E4 domains of LRP6 by phage antibody display using recombinant LRP6E1E2-Fc and LRP6E3E4-Fc fusions as the bait (Fig. 1a). Selection outputs were screened first against recombinant LRP6E1E2- and LRP6E3E4-Fc molecules by ELISA and then HEK293 cells transfected with full-length human LRP6 by FACS (Supplementary Fig. S1a). We identified specific binders and studied their abilities to regulate Wnt-induced β-catenin signaling using purified phage. Various effects were observed with some showing significant inhibition of the β-catenin-responsive luciferase SuperTopFlash (STF) reporter activity (Fig. 1b). Soluble recombinant scFv-Fc fusions derived from phage showed similar activities. For example, the E12N21 scFv against E1E2 and the E34N19 scFv against E3E4 domains of LRP6 were produced as scFv-Fc fusions and showed strong inhibition of Wnt1 and Wnt3a-induced STF reporter activity, respectively (Fig. 1c). We measured apparent binding affinities of E34N19 and E12N21 scFv-Fc molecules on HEK293 cells that express LRP6. The apparent K_D_ values were estimated to be 1.4 nM and 5.1 nM for the E34N19 and E12N21 scFv-Fc, respectively (Supplementary Fig. S1b and c). To determine their potency on Wnt-induced β-catenin signaling, HEK293 cells were transfected with plasmids encoding Wnt ligands (Wnt3a or Wnt1) and incubated with varying concentrations of scFv-Fc (E34N19 for Wnt3a or E12N21 for Wnt1). As shown in Fig. 1d, the scFv-Fcs inhibited β-catenin signaling with IC50 values of approximately 3.0 nM (for E34N19 scFv-Fc) and 3.5 nM (for E12N21 scFv-Fc). Thus we have identified anti-LRP6 human monoclonal antibodies with low nanomolar apparent binding affinities that inhibit LRP6E1E2/Wnt1- and LRP6E3E4/Wnt3a-mediated Wnt/β-catenin signaling with IC50 in the low nanomolar range.

### Construction of a bispecific antibody targeting LRP6 (the effector) and a tumor cell surface antigen (the guide)

**Figure 2:**
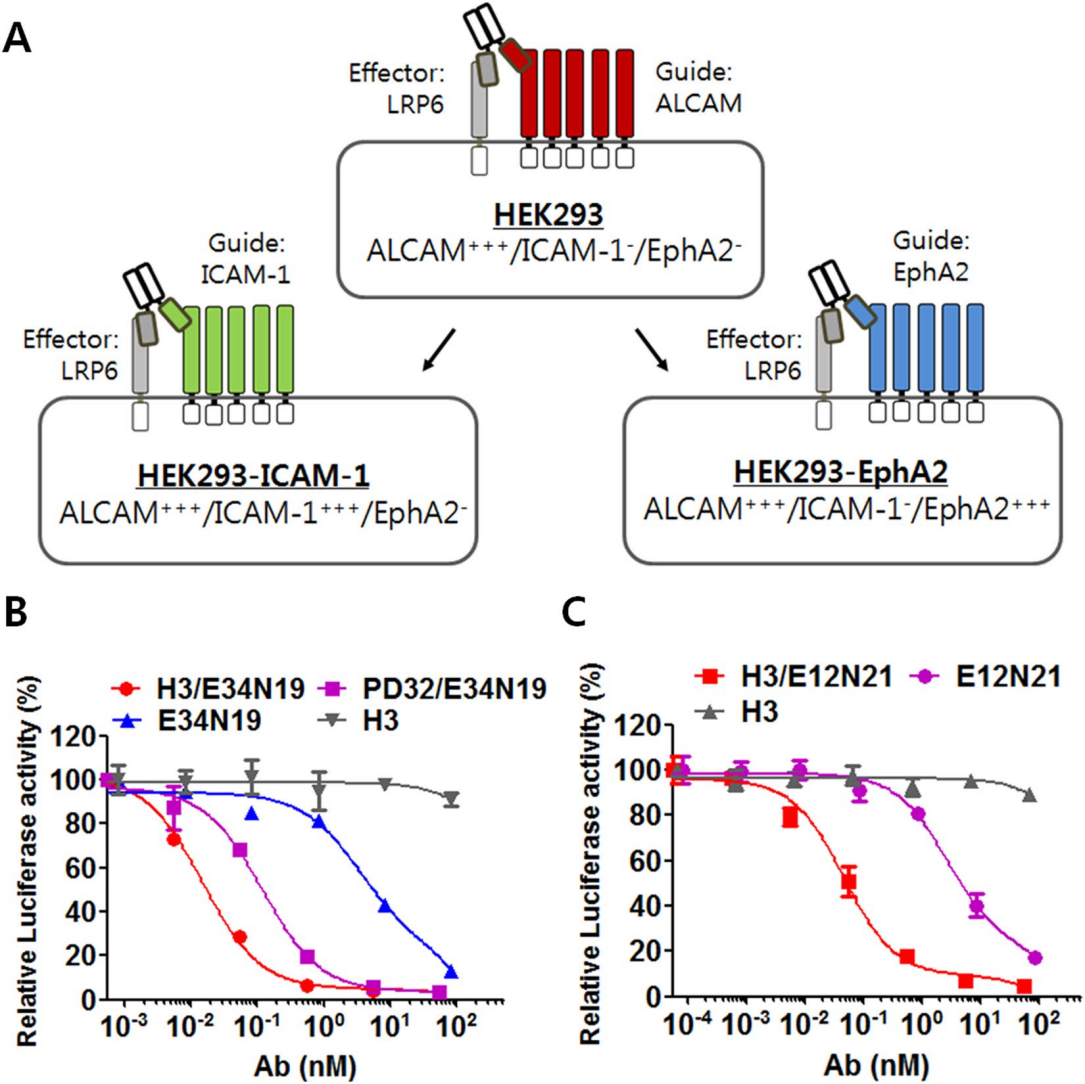
Guide/effector cell models and potent anti-Wnt signaling activity of guided anti-LRP6 bsAbs. (**a**) A schematic view of guide/effector cell models with different guide antigen expression levels. The HEK293-ICAM-1 (ICAM-1-positive) and HEK293-EphA2 (EphA2-positive) cells were generated using the parental HEK293 cell line (ALCAM-positive) by transient transfection or lentiviral transduction. (**b**) STF reporter assay on HEK293 cells transfected with Wnt3a-expressing plasmid and reporter constructs to assess potency of signaling inhbition by mono- and bi-specific anti-LRP6E3E4 antibodies. IC50 values are as indicated in the text (mean ± SD). (**c**) STF reporter assay on HEK293 cells transfected with Wnt1-expressing plasmid and report constructs. Relative luciferase reporter activities were separately analyzed. Values represent mean ± SD in duplicate determinations.

We hypothesized that a bispecific composed of an anti-effector (e.g., anti-LRP6) antibody and an antibody binding to a guide antigen (e.g., a tumor-associated cell surface molecule such as ALCAM, EphA2 or ICAM-1) will modulate potency and cell type selectivity of the anti-effector antibody. To test this hypothesis, we used HEK293-based cell models where the expression of guide antigens can be readily manipulated (Fig. 2a). We designed bispecific tandem scFv-Fcs (termed TaFv-Fc) composed of the anti-LRP6 E34N19 scFv and each of the following anti-guide scFvs: M10A12 (anti-ICAM-1), RYR (anti-EphA2), H3 (anti-ALCAM), and PD32 (anti-ALCAM that binds to an overlapping with H3 but with a reduced affinity) (Supplementary Fig. S2a). The TaFv-Fcs showed the expected molecular weight of about 90 kDa under reducing conditions (Supplementary Fig. S2a). To account for potential format differences, we also generated a bispecific IgG-scFv (bsIgG)^33^ by fusing anti-ALCAM H3 scFv to the light chain C-terminus of the anti-LRP6 E34N19 IgG (Supplementary Fig. S2b). We then tested binding by FACS of bispecific H3/E34N19 TaFv-Fc or E34N19/H3 bsIgG to HEK293 cells (Supplementary Fig. S2c), which express a low level of LRP6 but a high level of ALCAM (Supplementary Table S1). As expected, the bispecific H3/E34N19 TaFv-Fc and E34N19/H3 bsIgG and the monospecific H3 IgG had high median fluorescence intensity (MFI) values on HEK293 cells, whereas the monospecific E34N19 scFv-Fc or IgG exhibited low MFIs (Supplementary Fig. S2c). The monospecific anti-ALCAM H3 IgG binds strongly to HEK293, consistent with a high level of ALCAM expression on those cells (Supplementary Fig. S2c). We tested all of our bispecific antibodies in this manner on cells expressing high levels of the appropriate tumor-associated guide antigens and confirmed the expected pattern and level of binding (similar to the monospecific guide antibody) (Supplementary Fig. S2d), indicating that the guide antibody component is functional in all our bispecific constructs.

### Bispecific anti-LRP6/anti-tumor antibody greatly enhances the potency of Wnt-signaling inhibition

We next performed STF reporter inhibition assays to determine if bispecific antibodies incorporating a guide antibody component are capable of more potent inhibition of Wnt signaling than their monospecific counterparts. Both bispecific TaFv-Fcs targeting LRP6 and ALCAM (H3/E34N19 and PD32/E34N19) showed significantly more potent STF reporter inhibition compared to that of monospecific E34N19 scFv-Fc with IC50 being 15.14 pM for H3/E34N19 and 118.8 pM for PD32/E34N19 compared to 3.43 nM for the monospecific E34N19 (Fig. 2b). The more potent inhibition by H3/E34N19 compared to PD32/E34N19, both of which bind to ALCAM, may be due to the higher affinity binding of the guide antibody H3 compared to PD32 (apparent K_D_ on cell being 7.34 pM for H3 and 387.5 pM for PD32). To investigate this effect, we measured the apparent affinity of the bispecific H3/E34N19 and PD32/E34N19 on HEK293 cells and found that the apparent affinity of H3/E34N19 is 20-fold higher than that of PD32/E34N19 (Supplementary Fig. S3a and b), consistent with the affinity differential seen in the corresponding mAbs. Since both the H3 and the PD32 guide antibodies bind to the same epitope on the same cell surface target (ALCAM), this suggests that the potency of Wnt signaling blockade by the bispecific is at least partially determined by the affinity of the guide antibody. The E34N19/H3 bsIgG, a different form of the bispecific, also showed a profound STF reporter inhibitory effect compared to the monospecific E34N19 IgG, suggesting that the observed potency enhancement applies across different bispecific platforms (Supplementary Fig. S3c). To broaden applicability of the bispecific-based potency enhancement, we created H3/E12N21 TaFv-Fc composed of the H3 scFv (anti-ALCAM) and the anti-LRP6E1E2 E12N21 scFv (Wnt1 blocker). H3/E12N21 was 50 times more potent in inhibiting Wnt1-induced reporter activity than the monoclonal E12N21 (IC50 of 46.16 pM vs. 2.63 nM) (Fig. 2c), suggesting that the observed potency enhancement is not unique to the E34N19 antibody but rather generally applicable.

### Cell type-selective Wnt signaling pathway inhibition by bispecific antibody

**Figure 3:**
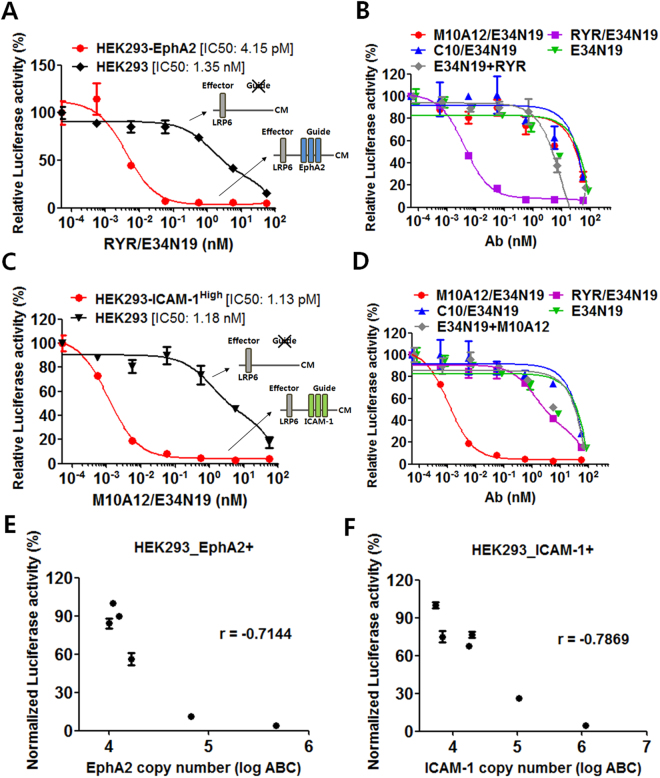
Anti-LRP6 bsAbs potently suppress Wnt/β-catenin reporter activity in a guide antigen-selective manner. (**a**) Selective Wnt3a/β-catenin signaling inhibition by EphA2-guided bispecific RYR/E34N19 on EphA2-expressing cells. HEK293-EphA2 cells were transfected with reporter and Wnt3a-expression constructs, and treated with varying concentrations of RYR/E34N19. The parental HEK293 cell line that expresses no/low EphA2 was treated in parallel to provide a control for cell type specificity. IC50 values are indicated on the graph (mean ± SD in duplicated experiments). The cartoons next to the curves indicate guide/effector configuration on HEK293 and HEK293-EphA2 cells. CM: Cell membrane. (**b**) Potent and cell-type selective signaling inhibition is unique to the bispecific but not antibody mix. HEK293 cells were transiently transfected with reporter plasmids, Wnt3a- and EphA2-expression constructs, and treated with indicated antibodies. E34N19 + RYR: a mix of anti-LRP6 antibody E34N19 and anti-EphA2 antibody RYR. C10/E34N19: a non-binding antibody C10-based anti-LRP6 bispecific that serves as a control. M10A12/E34N19: ICAM-1 guided anti-LRP6 bispecific, which serves as a control as HEK293 cells express no/low ICAM-1. (**c**) Wnt3a/β-catenin signaling blockade by ICAM-1-guided bispecific M10A12/E34N19 in ICAM-1 expressing cells. STF reporter assays were performed on HEK293 cells transiently transfected to express high levels of ICAM-1 (HEK293-ICAM-1^High^). The parental HEK293 cell line expressing no/low ICAM-1 was used as a control for specificity. (**d**) Potent and cell-type selective inhibition of Wnt3a/β-catenin signaling by M10A12/E34N19 is unique to the bispecific but not antibody mix. STF reporter assays were conducted as in (**c**) using indicated antibodies. E34N19 + M10A12: a mix of anti-LRP6 antibody E34N19 and anti-ICAM-1 antibody M10A12. C10/E34N19: a control bispecific (described in (**b**) above). RYR/E34N19: EphA2-guided anti-LRP6 bispecific, which serves as a control as HEK293 cells express no/low EphA2. (**e**) Impact of guide antigen density on Wnt/β-catenin reporter inhibition by EphA2-guided bispecific RYR/E34N19. HEK293 cells expressing varying copy numbers of cell surface EphA2 were prepared by transient transfection and treated with RYR/E34N19 (10 nM). (**f**) Impact of guide antigen density on Wnt/β-catenin reporter inhibition by ICAM-1-guided bispecific M10A12/E34N19. Each normalized reporter activity in (**e**) and (**f**) was plotted and Pearson’s correlation coefficient (r) was calculated from the graph. Error bars represent SD of a duplicate.

The guide antigen not only provides enhanced potency but more importantly cell type specificity of the signaling pathway inhibition. HEK293 cells express very low levels of ICAM-1 and EphA2 and are considered to be ICAM-1 negative/EphA2 negative. We first generated an EphA2-expressing HEK293 cell line (named HEK293-EphA2) by lentiviral transduction and used it to evaluate mono- or bi-specific antibodies in Wnt signaling blockade. As shown in Fig. 3a, inhibition of Wnt3a-induced signaling by the anti-EphA2/LRP6E3E4 (RYR/E34N19) bispecific is 325-fold more potent in the guide antigen expressing HEK293-EphA2 than the parental HEK293 cells (IC50 4.15 pM for HEK293-EphA2 vs. 1.35 nM for HEK293). The guide antigen dependence was further studied on HEK293 cells using control bispecific and monospecific antibodies. As shown in Fig. 3b, the pM inhibitory effect is only seen for the bispecific (RYR/E34N19) that targets both the guide and the effector antigens, but not for (1) the anti-effector mAb E34N19; (2) a simple mixture of mAbs that target the guide and the effector (RYR + E34N19); (3) a control bispecific that does not bind to the guide antigen, i.e., C10/E34N19 with C10 being a non-binding antibody, and M10A12/E34N19 with M10A12 binding to ICAM-1 that is not expressed by HEK293. To broaden applicability, we next studied cell-type specificity using a second guide antigen ICAM-1. We transfected HEK293 cells with ICAM-1 expressing plasmid to create guide antigen-positive cells. As shown in Fig. 3c, the bispecific M10A12/E34N19 is a 1,044-fold more potent inhibitor of Wnt signaling in HEK293-ICAM-1 (IC50 = 1.13 pM) compared to HEK293 (IC50 = 1.18 nM) cells. Control bispecific and monospecific antibodies were also studied (Fig. 3d). The striking potency enhancement is clearly dependent on binding to both the guide and the effector antigens, and is not observed for (1) the monospecific anti-effector antibody, (2) the simple mix of the monospecific anti-guide and anti-effector antibodies, or (3) control bispecific that does not bind to the guide antigen (C10/E34N19 and RYR/E34N19, neither of which binds to the guide antigen ICAM-1 or the HEK293-ICAM-1 expressing cells).

### The guide antigen expression level impacts the potency of the bispecific-mediated cell type-specific Wnt signaling inhibition

We sought to determine if the cell surface expression level of the guide antigen impacts the potency of the bispecific. As stated above, HEK293 cells express very low levels of ICAM-1 and EphA2, creating an opportunity to investigate the issue using HEK293 cells expressing varying levels of the guide antigen (ICAM-1 or EphA2). We first measured cell surface antigen copy number using a FACS-based quantification method that we used previously^34^ that determines the antibody binding capacity (ABC, operationally defined as “antigen density”) from median fluorescence intensity (MFI) (Supplementary Table S1). STF reporter assays were performed and IC50 values were determined for the M10A12/E34N19 bispecific on HEK293 cells that display varying guide antigen (ICAM-1) density: 484,486 (IC50 = 1.13 pM), 205,962 (IC50 = 3.76 pM), 30,702 (IC50 = 16.89 pM) and 9,011 (parent HEK293, IC50 = 1.18 nM) (Supplementary Table S2). (The monospecific E34N19 has IC50 = 3.02 nM as shown in Fig. 1d) A threshold level of ICAM-1 guide antigen expression (about 15,000–20,000 copies/cell) is required for significant enhancement of reporter inhibition. Above the threshold, the potency increases as the guide antigen density increases. The dependence on the guide antigen expression level was also shown in another assay where a given concentration of the bispecific was incubated with HEK293 cells expressing varying amounts of the guide antigen EphA2 or ICAM-1 (cell surface copy number measurement is shown in Supplementary Table S2). Pearson’s correlation coefficient analysis showed increased potency of reporter inhibition as the cell surface copy number of the guide antigen increases (Fig. 3e and f). The ratio of the guide to effector antigen density is also shown in Supplementary Table S2. Based on the three guide antigens that we studied, a guide to effector ratio >5–10 is generally required to achieve two to three orders of magnitude of potency enhancement.

The impact of the guide antigen density on the apparent K_D_ of the bispecific was also evaluated (Supplementary Table S2). For the ALCAM-guided bispecific, increasing guide antigen density concurs with increasing potency. For the ICAM-1-guided bispecific, apparent K_D_ values decrease with increasing guide antigen density, but the change is about 10-fold, not enough to account for the 1,000-fold enhancement in potency. Similar results were seen for the EphA2-guided bispecific. Other mechanisms beyond avidity-driven apparent K_D_ improvement may account for the bispecific phenomenon that we have observed. To understand this huge potency enhancement, we tested the hypothesis that the bispecific causes persistent occupation of the effector antigen due to the presence of the guide antigen. We studied LRP6 occupancy by the bispecific vs. monospecific on HEK293 cells that express both the guide (ALCAM) and the effector (LRP6). Compared to the bispecific H3/E34N19, more than 500-fold higher concentration of the monospecific E34N19 is required to occupy 50% of the effector LRP6 (Supplementary Fig. S3d). Thus, the occupancy data suggests that the affinity of the bispecific H3/E34N19 for LRP6 is more than 500-fold higher than the monospecific E34N19 on HEK293 cells expressing the ALCAM guide antigen, which is consistent with the potency increases observed.

Taken together, our data show that a bispecific antibody incorporating a guide antigen-targeting component can significantly enhance potency and specificity of Wnt pathway inhibition. For a given level of effector antigen expression (e.g., LRP6), the overall inhibition potency is affected by (1) the guide antigen density or the guide/effector ratio (preferably > 10:1), and (2) the affinity of the anti-guide antibody. The mechanism of action of the bispecific seems to lie in the higher affinity for the effector antigen due to the presence of the guide antigen on the same cell.

### Internalizing guide antibody inhibits multi-ligand Wnt signaling via cell surface removal of LRP6 (effector)

**Figure 4:**
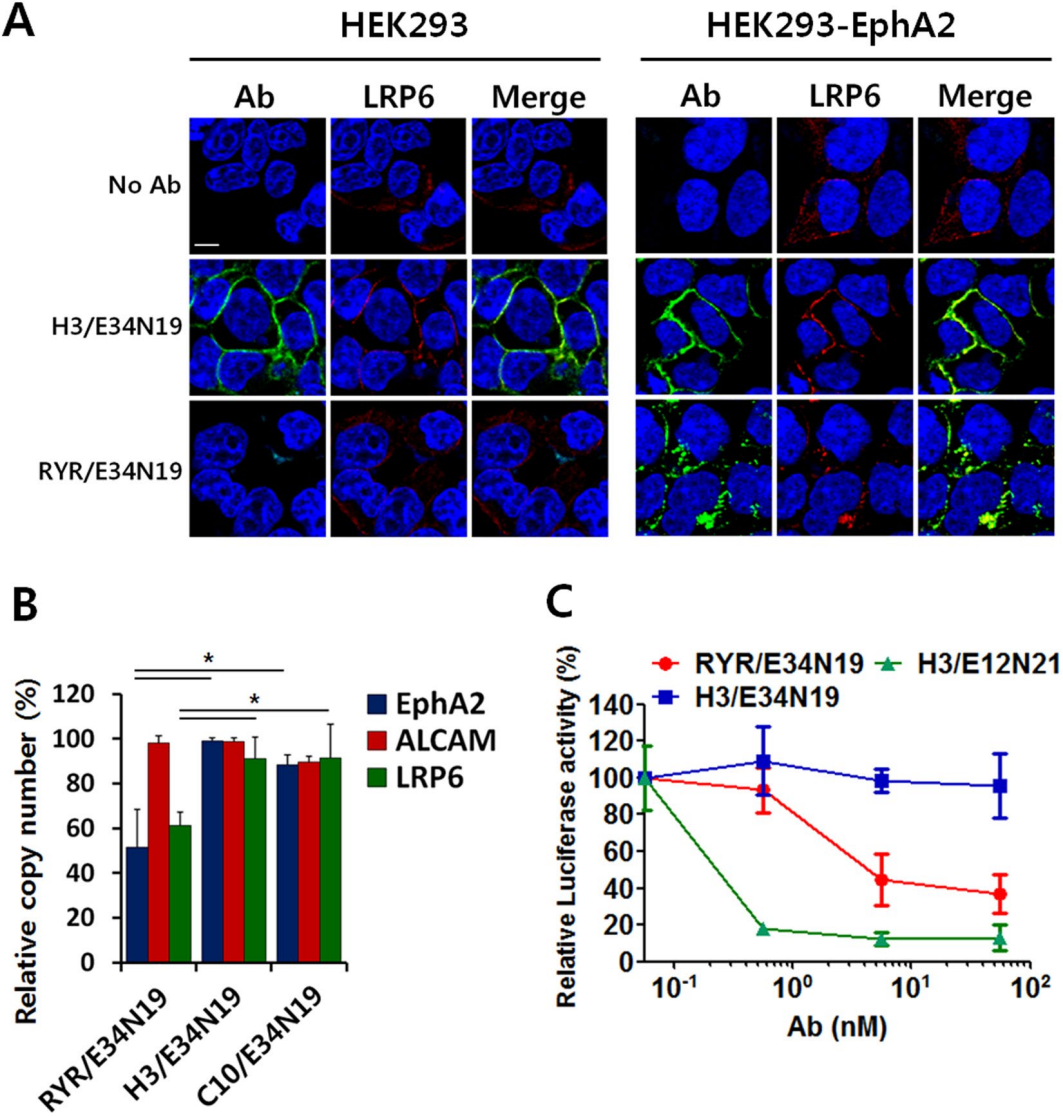
LRP6 internalization induced by RYR/E34N19 inhibits Wnt1-mediated β-catenin signaling. (**a**) LRP6 co-localization with internalizing vs. non-internalizing bsAbs. HEK293 and HEK293-EphA2 cells were incubated with the non-internalizing H3/E34N19 or the internalizing RYR/E34N19. Cell-associated antibodies were stained with secondary anti-human IgG-FITC (*green*), and LRP6 was detected by Alexa^®^ 647-labeled E12N21 IgG (*Red*). Stained cells were imaged under a confocal microscope. Scale bar, 10 μm. (**b**) Cell surface guide- and effector-antigen densities after bsAb-mediated internalization. HEK293-EphA2 cells were incubated with RYR/E34N19, H3/E34N19, or C10/E34N19 and washed to remove unbound antibodies. Copy numbers of EphA2, ALCAM, or LRP6 on cell surface were determined using separate Alexa^®^ 647-labeled antibodies that recognize a different epitope on each antigen. Calculated copy number (ABC) was normalized against an ABC value of each antigen measured on cells without antibody treatment. Data represent mean ± SD of a duplicate. *P < 0.05. (**c**) Inhibition of Wnt1-induced signaling by LRP6 turnover. HEK293-EphA2 cells were transfected with reporter and Wnt1-expression constructs, followed by treatment with varying concentrations of each indicated TaFv-Fc. Values represent mean ± SD for a duplicate. *P < 0.05.

We next studied if an internalizing guide antigen can cause cell surface removal of the effector antigen thereby inhibiting the Wnt signaling pathway. As previously reported by us^27^, the anti-EphA2 scFv (RYR) used in this study rapidly internalizes via macropinocytosis and efficiently removes EphA2 from the cell surface. We first studied internalization of the bispecific RYR/E34N19 on the EphA2-overexpressing HEK293-EphA2 cell line. RYR/E34N19 is internalized rapidly and specifically by HEK293-EphA2 cells, and it drags the effector antigen LRP6 together with the guide antigen EphA2 into the cells (Fig. 4a). In contrast, the H3/E34N19 bispecific, with H3 being a slowly internalizing antibody binding to the guide antigen ALCAM expressed by HEK293, is detected mainly on the cell surface with LRP6 (Fig. 4a). To evaluate quantitatively this target-specific internalization event induced by RYR/E34N19, we investigated the surface expression level of effector and guide antigens after incubating HEK293-EphA2 cells with bispecific antibodies. As shown in Fig. 4b, incubation of RYR/E34N19 efficiently reduced the cell surface copy numbers of both EphA2 (the guide) and LRP6 (the effector). No surface depletion was observed for either a bispecific antibody that binds to a slowly internalizing guide antigen (H3/E34N19) or a control bispecific that does not bind to any guide antigen expressed by the target cell (C10/E34N19) (Fig. 4b).

Surface removal of the LRP6 effector antigen should inhibit signaling by all LRP6-dependent ligands. We studied inhibition of Wnt1-mediated signaling through LRP6 internalization induced by the bispecific RYR/E34N19 that binds to the Wnt3a binding site on LRP6 (E3E4) and the internalizing guide EphA2. To begin, we first tested Wnt1 signaling inhibition by the bispecific built on the non-internalizing anti-guide antibody. On Wnt1-transfected HEK293-EphA2 cells, the ALCAM-guided H3/E12N21 that binds to the Wnt1 binding site but not H3/E34N19 that binds to the Wnt3a binding site potently inhibited STF reporter activity (Fig. 4c). This is expected as E12N21 but not E34N19 blocks Wnt1-mediated signaling. H3 binds to ALCAM that is non/slowly internalizing and does not result in LRP6 surface removal (shown in Fig. 4b). In contrast, the EphA2-guided bispecific RYR/E34N19 showed significant inhibitory effect on Wnt1-induced signaling, even though it binds to the E3E4 but not the E1E2 domain and does not directly interfere with Wnt1 binding to LRP6. This suggests that the RYR/E34N19 bispecific removed the effector antigen LRP6 from the cell surface and in so doing blocked Wnt signaling initiated by a ligand binding to a different region of the effector LRP6. Thus, a bispecific antibody constructed with an internalizing anti-guide antibody can induce receptor removal from cell surface and block receptor-dependent signaling induced by multiple ligands in a potent and cell-type selective manner.

### Application of the bispecific design to Wnt inhibition in tumor cells

Going beyond the HEK293 model system, we sought to determine if the principle of the bispecific guide/effector design is applicable to tumor cells. We first studied the A549 lung cancer cell line that expresses the effector LRP6 (7,341 copy/cell, Supplementary Table S1) and the guide ALCAM (874,813 copy/cell, Supplementary Table S1). Cell binding data for bispecific (H3/E34N19 and H3/E12N21) and monospecific (E34N19, E12N21, and H3) antibodies are shown in Supplementary Fig. S4a. As shown in Supplementary Fig. S4b and C, the bispecific H3/E34N19 and H3/E12N21 showed greater potency (>300-fold) in inhibiting the Wnt3a- or Wnt1-induced STF reporter activities, respectively, compared to that of the monospecific E34N19 or E12N21. We also studied receptor occupancy of the effector LRP6 by bispecific and monospecific antibodies under the same condition as that of the STF reporter assay. Similar to the result obtained from the HEK293 study (Supplementary Fig. S3d), we found that more than 300-fold higher concentration of the monospecific than the bispecific is required to achieve 50% occupation of the effector LRP6 (Supplementary Fig. S4d). Thus, similar to the results obtained with HEK293 cells, it appears that the affinity of the bispecific for LRP6 is >300-fold higher than monospecific.

**Figure 5:**
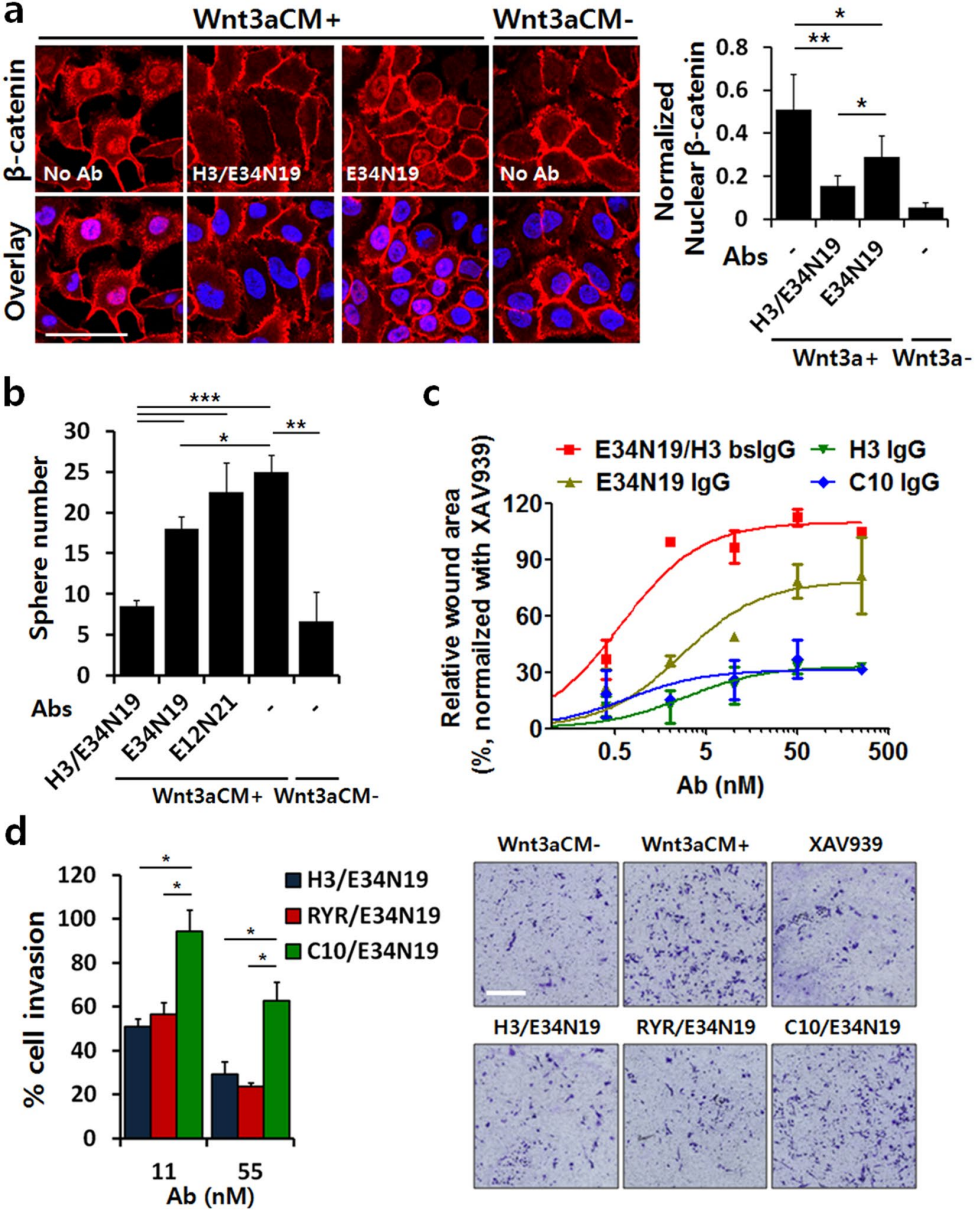
Guided anti-LRP6 bsAbs enhances anti-tumorigenic activities *in vitro*. (**a**) Immunostaining analysis assessing nuclear translocation of β-catenin. A549 cells were treated with 20% Wnt3a-CM and various antibodies (100 nM). Cells incubated without Wnt3a-CM and/or antibodies were used as controls. Cellular β-catenin (*red*) and nuclei (*blue*) were stained, and confocal images were overlaid to determine nuclear localization of β-catenin. Nuclear β-catenin signals were quantified using ImageJ and normalized with background fluorescence (*right*). Scale bar denotes 100 μm. *P < 0.05 and **P < 0.01. (**b**) Potent inhibition of tumor sphere-formation by H3/E34N19. Single cell population of A549 was incubated in sphere-forming media supplemented with or without 10% Wnt3a-CM and each antibody (50 μg/ml) for 2 weeks. The number of tumor spheres with > 100 μm in diameter was quantified from each well. Data represent mean ± SD of a duplicate. *P < 0.05, **P < 0.01, and ***P < 0.001. (**c**) Wound healing assay to assess inhibition of migration by bsAbs. A549 monolayer was scratched and treated with 20% Wnt3a-CM and varying concentrations of antibodies. Wound areas were quantified using ImageJ and normalized against a positive control (XAV939, 50 μM)^35^. Data represent mean ± SD of a duplicate. (**d**) Transwell assay to assess inhibition of cell invasion by bsAbs. PC3 cells seeded in the upper compartment of Transwell chambers were incubated with TaFv-Fc, with 20% Wnt3a-CM placed in the lower compartment. Invaded cells were stained with crystal violet after 24 h incubation and microscopic images were taken at 10x magnification. Scale bar: 200 μm. The number of invasive cells was normalized against a control group without antibody treatment (*left*). Representative images of stained Transwells are shown (*right*). *P < 0.05.

To expand the observation beyond reporter assays, we next studied if bispecific antibodies can affect cell function in a cell-type selective manner. We investigated: (1) effects on nuclear β-catenin levels in lung cancer A549 cells and found that the bispecific H3/E34N19 treatment significantly reduced β-catenin translocation into the nuclei, and the effect is more potent than the monospecific E34N19 (Fig. 5a); (2) effects on self-renewal capacity^9^ using tumor sphere formation assay on ALCAM^High^ cells (A549 and HEK293). The ALCAM-guided bispecific H3/E34N19 showed a stronger inhibitory effect on both A549 and HEK293 sphere formation induced by Wnt3a compared to the monospecific E34N19 (Fig. 5b and Supplementary Fig. S5a). Similar results were obtained on the ALCAM^High^ colon cancer cell line HCT116 but not the ALCAM^Low^ HT29 cells (Supplementary Fig. S5b; antigen density shown in Supplementary Table S1), consistent with the key role of the guide antigen in potent and specific Wnt signaling inhibition; (3) effects on tumor cell migration/invasion. In the scratch wound-healing migration assay, Wnt3a-induced A549 cell migration was more potently inhibited by the bispecific compared to the monospecific (Figs 5c and S6). In the invasion assay on PC3 that is ALCAM^High^ and EphA2^High^, both the ALCAM-guided H3/E34N19 and the EphA2-guided RYR/E34N19, but not the control bispecific C10/E34N19, effectively inhibited PC3 invasion (Fig. 5d); (4) effects on Wnt3a-dependent tumor cell (A549 and HCT116) proliferation. The bispecific H3/E34N19 is more potent compared to the monospecific E34N19 (Supplementary Fig. S7a for A549 and S7b for HCT116). Taken together, these results show that bispecific antibodies inhibit Wnt signaling in tumor cells, affecting β-catenin activity, sphere formation/clonogenic activity, migration/invasion, and proliferation of cancer cells *in vitro*, and the effect is enhanced in cells expressing the guide antigen.

### The bispecific does not affect wnt signaling in normal cells that express no or a low level of the guide antigen

To expand the study on cell type selectivity and functional specificity, we next investigated the effect of the bispecific on two normal cell lines MC3T3-E1 (a mouse preosteoblast line) and C3H/10T1/2 (a mouse mesenchymal stem cell line), which are commonly used in study of osteoblast differentiation controlled by Wnt signaling. We first showed that our anti-effector (E3E4N19 against LRP6E3E4) and anti-guide (RYR against EphA2) antibodies bind to both murine and human targets (Supplemental Fig. S8A and B). We next determined that both MC3T3-E1 and C3H/10T1/2 cells express a low level of the guide antigen EphA2 and has a low guide to effector ratio (Supplementary Fig. S8C and D). We then performed the STF reporter assay and found that the EphA2-guided RYR/E34N19 did not affect wnt signaling in both cell lines (Supplementary Fig. S8E and F), demonstrating that the bispecific is cell type selective and does not inhibit wnt signaling in normal cells that express no or a low level of the guide antigen.

### Anti-LRP6 bsAbs efficiently inhibits additional β-catenin activation induced by RSPO1

**Figure 6:**
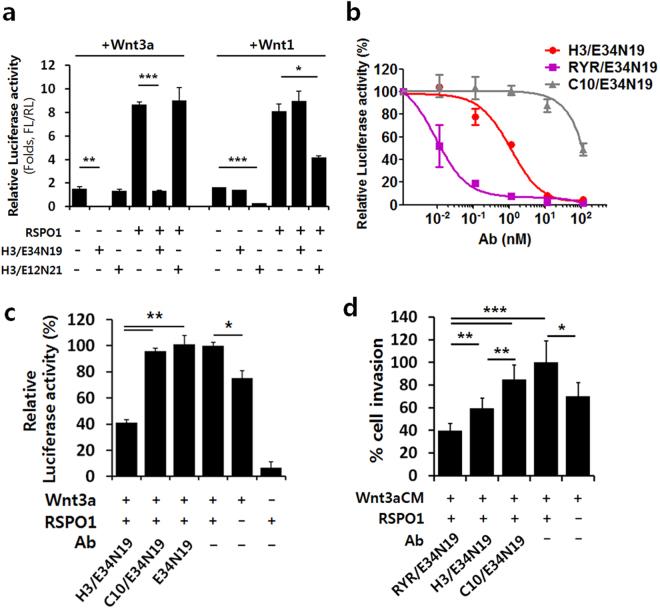
Guided anti-LRP6 bsAbs suppress β-catenin activity stimulated by RSPO1. (**a**) STF reporter assay assessing RSPO1-stimulated β-catenin activation and signaling inhibition by bsAbs. HEK293 cells transfected with STF reporter and Wnt3a- or Wnt1-expression constructs were treated with or without recombinant RSPO1 (100 ng/ml) and TaFv-Fc (10 μg/ml). Values represent mean ± SD of a duplicate. (**b**) Impact of antigen copy number on inhibition potency of RSPO1-induced Wnt3a/β-catenin signaling by bsAbs. HEK293-EphA2 cells that express different levels of the guide antigen EphA2 or ALCAM were transfected with STF reporter and Wnt3a-expression constructs, followed by treatment with recombinant RSPO1 (100 ng/ml) and varying concentrations of TaFv-Fc. Values represent mean ± SD for n = 3. (**c**) Potent inhibition of RSPO1-induced Wnt3a/β-catenin signaling by H3/E34N19 in A549 cancer cells. Cells were transfected with STF reporter and Wnt3a-expression constructs, followed by treatment with recombinant RSPO1 (100 ng/ml) and antibody (10 nM). Error bars represent SD (duplicate). (**d**) Anti-invasive effects of bsAbs under Wnt3a/RSPO1-induced signaling stimulation. PC3 cells seeded in the upper compartment of Transwell with antibody, with 20% Wnt3a-CM and recombinant RSPO1 (100 ng/ml) placed in the lower compartment. Invaded cells were visualized by crystal violet staining after 24 h incubation. The number of stained cells was normalized against a control group with no antibody treatment. *, **, and *** in graphs denote *P*-values of <0.05, <0.01, and <0.001, respectively.

It has been shown that RSPO1 stimulates Wnt/β-catenin signaling by preventing DKK1/Kremen- or RNF43/ZNRF3-mediated turnover of LRP6 on the cell surface^18,36^. To determine whether our guided anti-LRP6 bsAbs can antagonize RSPO1-stimulated Wnt signaling, HEK293 cells transiently expressing Wnt3a or Wnt1 ligand were incubated with RSPO1 and the ALCAM-guided bispecific H3/E34N19 that blocks the Wnt3a binding site or H3/E12N21 that blocks the Wnt1 binding site, respectively. In agreement with previously reported results^37^, RSPO1 amplified Wnt3a- or Wnt1-mediated reporter activity (Fig. 6a). Treatment with H3/E34N19 or H3/E12N21 led to potent inhibition of the reporter response induced by Wnt3a/RSPO1 or Wnt1/RSPO1, respectively (Fig. 6a). The IC50 values for the bispecific H3/E34N19 and the control bispecific C10/E34N19 are 327 pM and >100 nM respectively (Supplementary Fig. S9a). We further assessed cell type specificity of the bispecific effect on RSPO1-induced Wnt signaling, using the HEK293-EphA2 line that overexpress the guide antigen EphA2 (the parental HEK293 line expresses EphA2 poorly). Under conditions that support Wnt3a and RSPO1 signaling, the anti-EphA2/LRP6 bispecific (RYR/E34N19) showed a potent reporter inhibition in HEK293-EphA2 cells (IC50 = 9.0 pM, Fig. 6b), over 5,000-fold greater than that in parent HEK293 cells (IC50 = ~50 nM, Supplementary Fig. S9a). Moreover, on HEK293-EphA2 cells, RYR/E34N19 is 120-fold greater in potency than H3/E34N19 (IC50 = 9.0 pM vs. 1.1 nM as shown in Fig. 6b), due to the difference in guide antigen density (5,244,589 for EphA2 vs. 268,022 for ALCAM, Supplementary Table S1). EphA2 is also rapidly internalizing, which may have further enhanced the potency of the EphA2-guided bispecific compared to the non/slowly internalizing ALCAM-guided bispecific.

Going beyond the reporter assay on the HEK293 cell-based models and extending the finding to tumor cells, the bispecific phenomenon was further studied on the A549 cancer cell line. As shown in Fig. 6c, the Wnt3a/RSPO1-induced STF reporter activity was inhibited following treatment with the ALCAM-guided bispecific H3/E34N19 but not the control bispecific C10/E34N19 or the monospecific E34N19. Western blot analysis also showed that the levels of phosphorylated LRP6 and active β-catenin enhanced by RSPO1 were reduced significantly following H3/E34N19 treatment compared to controls (C10/E34N19 or E34N19 treatment) (Supplementary Fig. S9b).

To extend the finding to other tumor cells and to evaluate the impact of guide antigen internalization, we studied *in vitro* invasion assay in the presence of RSPO1 of prostate cancer PC3 cells that express similar levels of the non/slowly internalizing guide antigen ALCAM and the rapidly-internalizing guide antigen EphA2 (see Supplementary Table S1 for antigen density). As shown in Fig. 6d, both the ALCAM-guided bispecific H3/E34N19 and the EphA2-guided bispecific RYR/E34N19 inhibited PC3 cell invasion even in the presence of RSPO1. Interestingly, the EphA2-guided RYR/E34N19 that is internalizing showed a more potent inhibition than the ALCAM-guided H3/E34N19 that is non/slowly internalizing, suggesting that cell surface removal of LRP6 is an important factor in reducing RSPO1-stimulated Wnt signaling. These results suggest that bispecific anti-LRP6 antibodies can inhibit the RSPO1-mediated Wnt signaling amplification and downstream events such as tumor cell invasion, with the guide antigen providing cell type specificity and potency enhancement. Internalizing anti-guide antibody is more efficient in removing the effector antigen from the cell surface, resulting in greater potency compared to a non-internalizing bispecific.

## Discussion

Signaling pathways mediate cell homeostasis and are often used by both normal and diseased cells. Cell-type selective signaling pathway modulation, if achieved, will expand therapeutic window and enable development of effective therapy against cancer and other diseases. In this report, we describe a bispecific antibody-based approach that allows potent and specific signaling pathway inhibition initiated at the cell surface. We used the Wnt signaling pathway as a model system. We constructed a bispecific with one specificity targeting a cell-type selective antigen (the guide antigen) and the other the signaling receptor (the effector antigen, LRP6 for canonical Wnt signaling). We found that this guide/effector design selectively enhances signal pathway inhibition in target cells expressing the guide antigen. The potency of enhancement is striking for the bispecific, which is several hundreds to a thousand-fold more potent than the monospecific antibody. This phenomenon was observed using three different guide antigens (ALCAM, ICAM-1 and EphA2) and multiple types of effector-ligand interaction, i.e., Wnt3a and Wnt1 binding to LRP6E3E4 and E1E2 domains, respectively, as well as RSPO1-mediated Wnt signaling amplification. The results apply to not only model systems where HEK293 was engineered to express the guide antigen but also to a panel of tumor cell lines expressing varying amount of guide and effector antigens. Key parameters for the guide/effector design include (1) co-presentation of the guide and effector on the same cell (target cell), and a high ratio of the guide to effector antigen density. The potency of signal pathway modulation increases with increasing guide to effector ratio. Typically when the ratio is greater than 5–10:1, about a thousand-fold potency enhancement can be achieved in a target cell-specific manner. (2) If the guide antigen is internalized efficiently upon antibody binding (e.g., EphA2), the effector antigen (LRP6) can be removed from the cell surface by the bispecific, resulting in multi-ligand blocking (e.g., a bispecific targeting EphA2 and LRP6-Wnt3a binding site E3E4 can block Wnt1-mediated signaling that engages a different site E1E2). (3) A threshold level of cell surface guide antigen density is required to achieve the cell-type selective potency enhancement. For the three guide antigens that we studied, the level is about 15,000–20,000 per cell. The exact threshold of the guide antigen density may vary depending on the nature of the signaling pathway. In any event, when the guide antigen is expressed at a high level, the effect of the guided bispecific is apparent. This fits well with tumor targeting as many cell surface molecules have been found to be overexpressed by tumor cells, thus can serve as the guide antigens to redirect and enhance the activity of anti-LPR6 antibodies in Wnt signaling inhibition.

The guide antigen density impacts the behavior of the bispecific in two ways as shown by our study: (1) The apparent binding affinity of the bispecific to the target cell increases as the guide antigen density increases. The concurrent increase, however, is insufficient to explain the huge IC50 change seen for the bispecific. For example, for the ICAM-1-guided bispecific, the improvement of apparent affinity to target cell is modest (about 10-fold), but the IC50 value decreases by over 1,000-fold compared to the monoclonal anti-effector antibody. (2) The guide antigen density dramatically impacts the apparent affinity of the bispecific to the effector antigen as measured by the occupancy study. When the ratio of the guide to effector antigen density is greater than 10 to 1, binding of the bispecific resulted in almost complete occupation of the effector antigen at concentrations well below the apparent K_D_ of the bispecific to the target cell. The guide antigen seems to provide a local or community effect where the effector antigen is persistently occupied even when the guide antigen itself is only partially occupied. This community effect is only seen when the guide and effector antigens are present in cis on the same target cell, and is unique to the bispecific but not a mix of monoclonal antibodies (i.e., oligoclonal antibodies).

Bispecific antibodies have emerged as a new class of biologic with promise in clinical applications. Various forms of bispecific antibodies have been developed^22,25,31^. Catumaxomab, a rat-mouse hybrid targeting epithelial cell adhesion molecule (EpCAM) and CD3, and blinatumomab, a mouse antibody targeting CD19 and CD3, have been approved by the FDA for treating malignant ascites and acute lymphoblastic leukemia, respectively. In both cases, the bispecific engages two targets on two different cell types (tumor and effector cells), with the goal of bringing the effector to the tumor cell for killing. Most of the bispecific under clinical development follows this type of design. Our bispecific, however, differs from those traditional bispecific as in our guide-effector system, the guide and effector antigens are co-presented on the same target cell. When a set of conditions is met, i.e., guide to effector ratio and threshold level of guide expression, our bispecific can achieve a striking synergistic effect on the effector antigen-mediated signaling. We studied two different bispecific formats, tandem scFv-Fc and IgG-scFv, and found that the bispecific phenomenon was observed for both, suggesting that our guide/effector design is not format-bound and should be applicable to other forms of bispecific.

Our guide/effector bispecific design enables cell-type selective signaling pathway modulation, and should facilitate development of new therapies that more selectively and potently inhibit signaling pathways critical for tumorigenesis. Our study is focused on Wnt signaling pathway as a model system, but the approach has potential to be generally applicable. A notable difference between our guide/effector approach and other co-targeting strategies is that the latter is based on the idea of co-blocking of two signaling pathways (e.g. EGFR and IGF1R)^22^. Toxicity could be a concern there as both antibodies bind to signaling receptors critical for cell growth and survival. Our approach allows modular development of bsAb with the goal of achieving maximum effect on tumor but minimum effect on normal cells. We chose ALCAM, ICAM-1 and EphA2 as model guide antigens to illustrate the principle. The threshold effect on each guide antigen suggests that there is no need for absolute specificity as the system can tolerate some level of normal tissue expression of the guide antigen. Depending on the nature of the effector antigen and the specific disease application, other tumor-specific or cell type-selective cell surface molecules can be used as guide antigens. We have made a novel finding regarding the relationship between the cell surface guide antigen density and the potency and specificity of the bispecific. Consistent with our finding, recent studies also pointed to an antigen density effect on bispecific antibodies using the EGFR/c-Met model although only a mild synergistic effect was observed^38,39^. The study was designed along the line of co-blocking two signaling pathways, and unlike our approach, there is no delineation of the guide and the effector.

Cell surface Wnt receptors in canonical Wnt signaling pathway, including Frizzled (Fzd) family members and LRP5/6, play an important role in cancer progression, especially in maintaining metastatic and self-renewal potential^9,40,41^. Anti-tumor activities have been observed using strategies that antagonize the interplay of Wnt-related receptors and ligands, including the use of mAbs against LRP6, Wnt1, Wnt2, Fzd7, Fzd10, or LGR5^19,42–46^ and Wnt-binding decoy receptors^47–51^. In agreement with these reports, we showed that our anti-LRP6 bsAbs negatively modulate proliferation, migration/invasion, and self-renewal activities in various cancer cell lines that utilize the Wnt pathway. Interestingly, unlike HEK293 cells where the STF reporter activity can be inhibited fully by anti-LRP6 antibodies (mono or bispecific, with the difference in IC50 but not maximum level of inhibition), we observed about 50% suppression of Wnt/β-catenin reporter activity in cancer cells. This is not due to lack of occupancy of the effector (LRP6), as we measured occupancy and found that in the case of the bispecific, the effector is nearly fully occupied at less than 100 pM of the bispecific. These results imply that cancer cells control the β-catenin activity by LRP6-independent Wnt signaling through other Wnt ligand/receptor interactions^7,52,53^. For example, the atypical receptor tyrosine kinase RYK as a Wnt co-receptor interacts with Wnt3a for β-catenin stabilization in osteosarcoma cells^54^, and the Fzd5 receptor cooperates with Wnt7a to activate the β-catenin-mediated STF signaling in ovarian cancer cells^55^. In addition, there are some interactions of Fzd receptors and/or ROR1/2 (receptor tyrosine kinase-like orphan receptor 1/2) with non-canonical Wnt ligands in Wnt-dependent cancer progression^52^. Thus additional blockade of LRP6-independent Wnt signaling should be applied for maximal Wnt signaling inhibition in cancer cells. This could be addressed by more complex strategies such as a combination treatment with multiple Wnt-inhibitory antibodies to block both the canonical and non-canonical signaling pathways.

In summary, our study suggests that cell type-specific, highly potent signaling pathway modulation can be achieved by a bispecfiic antibody that co-engages a cell type-selective antigen (guide) and the signaling receptor (effector) on the target cell. The potency and selectivity are modulated by the guide antigen density, the guide to effector ratio, and guide antigen-induced effector antigen internalization that removes the effector antigen from the cell surface. While our study is focused on the Wnt pathway, the approach should be generally applicable to cell-type specific modulation of signaling pathways cascading down from the cell surface.

## Methods

### Cell Lines

Human embryonic kidney (HEK) 293 and 293 A cell lines, lung cancer cell line A549, colon cancer cell lines HCT116 and HT29, prostate cancer cell line PC3, mouse preosteoblast cell line MC3T3-E1, mouse mesenchymal stem cell line C3H/10T1/2, and mouse CT26 were obtained from American Type Culture Collection (ATCC). Cells were maintained in DMEM, McCoy’s 5 A (colon cancer cell lines), or alpha-MEM (MC3T3-E1) supplemented with 10% FBS (Fisher Scientific), 100 μg/ml penicillin/streptomycin (Axenia BioLogix) at 37 °C in a humidified atmosphere containing 5% CO_2_.

### Plasmids

Full-length human LRP6, ICAM-1, or EphA2 cDNA cloned into pCMV-Entry (Origene) were used for sub-cloning or transient transfection. pFUSE-hIgG1-Fc2 (InvivoGen) was used for Fc-fusion constructs of truncated LRP6E1E2 and LRP6E3E4 domains. For bispecific tandem scFv-Fc constructs, pFUSE-hIgG1-Fc2 vector was modified by inserting a (G_4_S)_5_ linker between two scFv fusions. IgG-Abvec plasmids (Ig-γ and Ig-λ) were kindly provided by Dr. Patrick Wilson at University of Chicago^56^. The β-catenin-responsive luciferase reporter SuperTopFlash (STF) and the internal control pRL-SV40 Renilla luciferase constructs (Addgene) were used for STF reporter assays. To provide Wnt ligands in STF reporter assays, pcDNA-Wnt1 or -Wnt3a expression plasmid (Addgene) was utilized for transient transfections.

### Generation of Anti-LRP6 scFv Antibodies

Non-immune phage antibody display libraries from pooled healthy donors were used for antibody selection^34^. Recombinant LRP6E1E2 or LRP6E3E4 Fc-fusion was purified from HEK293A transfectants and used for selection rounds. Briefly, LRP6E1E2 or LRP6E3E4 Fc was coated on SPHERO^TM^ Polystyrene Magnetic Particles (Spherotech) by overnight incubation at 4 °C. Magnetic beads coated with each Fc fusion were incubated with phages that had been pre-depleted on uncoated beads for 1 h. Beads were then washed with PBS/0.1% Tween20 and PBS five times each, and bound phages were recovered and propagated as described previously^34,57–59^. After the third round of panning, monoclonal phage binders were screened by flow cytometry using full-length LRP6-transfected HEK293 cells. To detect phage binders, biotin-labeled anti-fd phage antibody (Sigma Aldrich) and streptavidin-R-PE (Invitrogen) were used as described^27^. To test the anti-Wnt signaling activity of phages by STF luciferase reporter assays, candidate phage clones were individually amplified and purified as previously described^29,58,60–62^.

### Recombinant Antibody Expression and Purification

To construct bispecific tandem scFv-Fc fusions, a nucleotide cassette consisting of restriction enzyme sites and a linker [AgeI-SalI-(Gly4Ser)_5_-NcoI-NotI] was inserted into pFUSE-hIgG1-Fc2 to create the pFUSE-T-scFv-Fc vector. AgeI and SalI or NcoI and NotI restriction enzyme sites were used for cloning of the anti-guide or the anti-effector (LRP6) scFv, respectively. To produce monospecific scFv-Fc fusions, scFv genes were cloned into the original pFUSE-hIgG1-Fc2 plasmid. To construct IgG, heavy and light chain variable fragments were amplified by PCR and subcloned into Ig-γ and -λ expression vectors, respectively, as described previously^34,56^. To construct IgG-scFv bispecific antibodies, the Ig-λ expression vector was modified to bear a (Gly4Ser)_3_ linker and restriction enzyme sites for cloning of the anti-guide scFv to the C-terminus of the light chain constant region. The anti-LRP6 IgG was used as the backbone, and the anti-guide scFv H3 was inserted into the modified Ig-λ expression vector to form VL-CL-(Gly4Ser)_3_-scFv^33^. To produce proteins from all constructs described above, plasmid DNA was mixed with polyethylenimine in Opti-MEM (Life Technologies) and added to HEK293A cells for 24 h. After changing media to serum-free DMEM containing Nutridoma-SP (Roche), the cells were cultured for 6–8 days. Supernatants containing secreted antibodies were collected and purified on protein A agarose (Pierce^®^/Thermo Scientific) following the manufacturer’s instructions. All purified antibodies were analyzed on SDS-PAGE gradient gels (4–20%) and stained with PageBlue^TM^ Protein Staining Solution (Thermo Scientific).

### SuperTopFlash (STF) Luciferase Reporter Gene Assays

HEK293 or A549 cells seeded in a 24-well culture plate were transfected with 200 ng/well of STF luciferase reporter and 20 ng/well of pRL-SV40 mixed with 1.5 μl/well TransIT-2020 (Mirus Bio), with or without plasmids encoding the Wnt1 or Wnt3a gene. Varying amounts of ICAM-1 or EphA2 plasmid DNA were transfected into HEK293 to create a graded expression of guide antigens. Six (HEK293) or 24 h (A549) post transfection, cells were treated with testing agents (purified phages or antibodies) diluted in growth media and further incubated for an additional 16 h. Firefly luciferase (FL) and Renila luciferase (RL) activities were separately measured using Dual-Luciferase Reporter Assay System (Promega) and each FL value was normalized with the corresponding RL value. Normalized data were expressed as a percentage relative to the control experiment without antibody treatment. To evaluate EphA2-targeted bispecific antibodies, HEK293-EphA2 cell model that stably expresses high levels of EphA2 was generated by lentiviral transduction and STF reporter assays were performed as described above. For RSPO1-mediated reporter activity stimulation, 100 ng/ml of recombinant human RSPO1 (R&D Systems) was to the culture medium in addition to ligands, and STF report assays were performed as described above.

### Antigen Copy Number Quantification

Cells were harvested by trypsinization, washed and resuspended in FACS buffer (PBS, 1% FBS, pH 7.4). Cell surface antigen density (copy number per cell) measurement was performed as described^34^. Briefly, primary anti-guide or effector antibody was fluorophore-labeled using the Alexa Fluor^®^ 647 Monoclonal Antibody Labeling Kit (Molecular Probes). We measured the effective Fluorophore/Protein (F/P) ratio of each Alexa Fluor^®^ 647-conjugated antibody using Simply Cellular^®^ anti-Human IgG (Bang’s Laboratory) according to manufacturer’s instructions. Following incubation with labeled antibody and PBS washing, cells were analyzed by BD Accuri C6 flow cytometer (BD Biosciences). MFI (Median Fluorescence Intensity) values were converted into Molecules of Equivalent Soluble Fluorochrome (MESF) using Quantum™ Alexa Fluor^®^ 647 MESF (Bang’s Laboratory) according to manufacturer’s recommendations. MESF was converted into Antibody Binding Capacity (ABC) using the F/P ratio determined above^34^.

### LRP6 Occupancy Quantification

Receptor occupancy of effector antigen LRP6 was determined after pre-treatment of target cells by mono- or bi-specific scFv-Fc constructs. A549 cells were incubated with varying concentrations of antibodies at 37 °C for 16 h, washed twice with chilled PBS, blocked with PBS supplemented with 1% BSA, and further incubated with Alexa Fluor^®^ 647-conjugated E34N19 IgG for 1 h on ice. The unoccupied LRP6 level was detected by flow cytometry. MFI values measured from each experimental group were normalized against the maximum MFI signal in the control group without antibody treatment.

### Apparent K_D_ Determination

The apparent antibody binding affinity was measured on target cells by FACS as described^28,60,61^. Briefly, 3 × 10^5^ cells were incubated with varying concentrations of antibodies in final 1 ml of FACS buffer at 4 °C overnight, washed with ice-cold PBS, resuspended in 100 µl FACS buffer containing Alexa Fluor^®^ 647-labeled goat anti-human IgG, incubated for 1 h at 4 °C, and analyzed by FACS. The apparent K_D_ was determined by curve fitting (GraphPad) using MFI values as described^60,61^.

### Immunofluorescence Confocal Microscopy Study

Cells were seeded at 1.5 × 10^4^/well on Labteck 8-well culture chamber slides (Thermo Fisher Scientific). To evaluate antibody-target antigen (LRP6) colocalization, cells were incubated with each antibody for 1 h in growth media, fixed with 4% PFA (paraformaldehyde), permeabilized in PBS/1% FBS/0.2% Triton-X100, and further incubated with FITC-labeled goat anti-human IgG for antibody detection and Alexa Fluor 647^®^-labeled anti-LRP6 IgG for LRP6 staining. To study nuclear β-catenin translocation, cells were seeded in chamber wells as described above, incubated with the testing antibody in DMEM/10% FBS supplemented with 20% Wnt3a conditioned medium (Wnt3a-CM) for 24 h, stained with mouse anti-β-catenin primary antibody (Cell Signaling Technology) for β-catenin detection, followed by Alexa Fluor^®^ 647-labeled rabbit anti-mouse secondary IgG (Jackson ImmunoResearch). All cells were counterstained using Hoechst33342 (Thermo Scientific) and imaged on FluoView^®^ FV10i laser confocal microscope (Olympus) with an Olympus 60X phase contrast water-immersion objective.

### Cell Proliferation Assays

A549 cells were seeded at 1.0 × 10^3^/well in 96-well cell culture plates, cultured overnight, and incubated with varying concentrations of antibodies diluted in DMEM/0.5% FBS/5% Wnt3a-CM at 37 °C for 96 h. Cell viability was determined using the Calcein-AM cell viability assay kit (Biotium).

### Immunoblotting

Whole cell lysates or cell-membrane fractions were prepared using RIPA buffer (50 mM Tris, pH 7.5, 150 mM NaCl, 0.1% SDS, 0.5% sodium deoxycholate, 1% NP-40) supplemented with protease/phosphatase inhibitors (Cell Signaling Technology). For cytosolic protein extraction, cells were resuspended in hypotonic buffer (10 mM Tris-HCl, 10 mM KCl, protease inhibitors, pH 7.5) and lysed by three freeze-thaw cycles. Equal amounts of protein were loaded onto a SDS-PAGE gradient gel (4–20%), electrophoresed, and electro-transferred to PVDF membrane (Millipore). Target proteins were probed with appropriate primary antibodies followed by incubation with secondary antibodies conjugated with Horseradish Peroxidase (HRP). Chemiluminescence signals were generated using ECL reagents (Millipore) and detected by c-DiGiT Blot Scanner (LI-COR). The blot image was analyzed using ImageJ^63^ to quantify band intensity.

### Cell Migration and Invasion Assays

For the wound-healing migration assay, cells were seeded in 96-well plates and allowed to reach confluence. Growth media was then replaced by serum-free media containing 10 μg/ml mitomycin C (Sigma Aldrich) and incubated for 2 h. A scratch was made on a uniform layer of cells using a sterile micropipette tip. After washing with PBS, cells were incubated with varying concentrations of antibodies diluted in DMEM/20% Wnt3a-CM for up to 48 h and stained with 2 μM Calcein-AM (Sigma Aldrich). The plate was scanned by CellInsight™ NXT HCS platform (Thermo Scientific) at indicated time points. Wound areas were measured using ImageJ software and IC50 values were generated based on the relative wound area data against the control group. For invasion assay, cells were assessed in Transwell with CultureCoat 24-well high BME insert (Trevigen). Cells resuspended in serum-free media with different concentrations of antibodies were added into the upper compartment of a Transwell insert. The culture medium containing 20% Wnt3a-CM was used as a chemoattractant and placed in the lower compartment. After 24 h incubation, the cells were fixed with 4% PFA and stained with 0.1% crystal violet (Sigma Aldrich) in PBS. The invaded cell number in each Transwell was counted in five fields spanning the membrane.

### Self-renewal Sphere-Forming Assay

For analysis of sphere formation, second-generation spheres were thoroughly trypsinized and sieved through a cell strainer with 40-μm nylon mesh (Thermo Fisher) to prepare a single-cell suspension. Cells were suspended in serum-free media (SFM) composed of DMEM/F12 (Gibco), 20 ng/ml EGF, 10 ng/ml bFGF, 10 ng/ml IGF, 2% B27 supplement (Gibco), and 10% Wnt3a-CM, and 200 cells per well were seeded in ultra-low attachment 24-well culture plate (Corning). Varying concentrations of antibodies were added and the cells were fed twice a week by 100 μl SFM. After 14-day incubation, 63 fields in each well were scanned and merged using BIOREVO microscope (BZ-9000; Keyence) to display the whole well image. The number of tumor spheres (>100 μM) was counted or measured from the images. To determine total number of viable sphere-forming cells, spheres were collected by centrifugation and dissociated into single-cell suspension by trypsinization. Cells were washed twice with PBS, stained with 2 μM Calcein-AM for 1 h, and analyzed by flow cytometry.

### Statistical analysis

All statistical analyses were conducted using GraphPad Prism 6.0 (GraphPad, Inc). For two group comparisons, P values were determined using two-tailed student’s T-tests and p < 0.05 was used to reject the null hypothesis. Correlation between luciferase reporter activity and guide antigen density was determined using Pearson’s correlation coefficient analysis. For multiple group comparisons, One-Way ANOVA was used using the Tukey’s test.

### Data availability statement

Supplementary data set is included in the submission. Any additional data related to this work is available upon request.

## Electronic supplementary material

Supplemental materials
