# Supplementary material for: Cell-type specific potent Wnt signaling blockade by bispecific antibody
Source: Sci Rep. 2018 Jan 15;8:766. doi: 10.1038/s41598-017-17539-z (PMC5768681; doi:10.1038/s41598-017-17539-z)
Supplement: Supplementary file 1 — Supplemental materials [file 41598_2017_17539_MOESM1_ESM.pdf]

Cell-type specific potent Wnt signaling blockade by bispecific antibody

Nam-Kyung Lee, Yafeng Zhang, Yang Su, Scott Bidlingmaier, Daniel W. Sherbenou, Kevin D. Ha, and Bin Liu

Supplemental Table 1. Summary of cell surface antigen density measurement.

Supplemental Table 2. Summary of guide antigen density, guide/effector ratio, apparent K_D and anti-Wnt signaling activity (IC50) for the guided bsAb.

Supplemental Fig. 1. Characterization of anti-LRP6 scFvs and derived bsAbs.

A) Anti-LRP6 scFv-phage screening by FACS. Individual scFv-phage clones were amplified from the 3rd round panning output against LRP6E1E2 or LRP6E3E4 domain. ScFv-phage particles were incubated with parental HEK293 or LRP6-transfected HEK293 cells and detected by biotinylated anti-M13 IgG and PE-labeled streptavidin. Relative MFI values (folds of phage binding to LRP6+ cells against LRP6- cells) were plotted on a graph. B and C) FACS-based K_D measurement of E34N19 (B) and E12N21 (C) scFv-Fc binding to HEK293 cells. The apparent K_D was calculated by curve fittings.

Supplemental Fig. 2. Generation and characterization of bsAbs.

A) Bispecific TaFv-Fc constructs. Purified E34N19 scFv-Fc (~ 60 kDa) and TaFv-Fc (~ 90 kDa) fusions were analyzed by SDS-PAGE under reducing condition. B) BsIgG

constructs. The anti-ALCAM H3 scFv is fused to the C terminal of E34N19 light chain. Under reducing conditions, SDS-PAGE analysis of E34N19 IgG shows two separate heavy (~50 kDa) and light (~25 kDa) chains, while E34N19/H3 bsIgG displays two bands, both at around 50 kDa. C) Cell-binding by mono- or bi-specific anti-LRP6 antibodies. Binding of E34N19 scFv-Fc, E34N19 IgG, H3/E34N19 TaFv-Fc, E34N19/H3 bsIgG, or H3 IgG (each 100 nM) to HEK293 that expresses ALCAM was analyzed by FACS. D) Binding specificity of TaFv-Fcs with different guide antibodies. HEK293 cells were transiently transfected with ICAM-1- or EphA2-expression construct, followed by incubation with each guided anti-LRP6 bsAb. Parental HEK293 cells express the guide antigen ALCAM only and were used as a specificity control for the EphA2-targeting RYR/E34N19 or the ICAM-1 targeting M10A12/E34N19 TaFv-Fc.

Supplemental Fig. 3. Enhanced cell binding and Wnt signaling inhibition by guided anti-LRP6 bsAbs.

A and B) Affinity measurement of bispecific anti-LRP6 TaFv-Fcs. HEK293 cells were incubated with varying concentrations of H3/E34N19 (A) or PD32/E34N19 (B) TaFv-Fc and analyzed by FACS. Apparent K_D values were calculated by curve fitting. C) STF reporter assay. E34N19 IgG and E34N19/H3 bsIgG were evaluated for inhibition potency of β-catenin reporter on Wnt3a-transfected HEK293 cells. IC50 was estimated by curve fitting (E34N19 IgG: 5.2 nM; E34N19/H3 bsIgG: 44.6 pM). Values represent mean ± SD for n = 3. D) Occupancy of the effector antigen LRP6 by targeting antibody. HEK293 cells were incubated with varying concentrations of E34N19 scFv-Fc or H3/E34N19 TaFv-Fc for 16 h. Cells were collected and washed with ice-chilled PBS three times to

remove unbound antibodies. Cell surface LRP6 levels were determined by binding of Alexa Fluor® 647-labeled E34N19 IgG to cells on ice. MFI values were normalized against a control group without antibody incubation. Values represent mean ± SD of a duplicate.

Supplemental Fig. 4. Enhanced cell binding and anti-Wnt signaling potency of bsAbs on A549 cancer cells.

A) Binding of H3 IgG, H3/E12N21, H3/E34N19 TaFv-Fc, E12N21 or E34N19 scFv-Fc (each 100 nM) to A549 cells that express ALCAM. B and C) STF reporter assays on A549 cells. Cells were transfected with STF reporter and Wnt3a- (B) or Wnt1-expression (C) constructs, followed by treatment with varying concentrations of H3/E34N19 vs. E34N19 (B), and H3/E12N21 vs. E12N21 (C), respectively. IC50 values were estimated from curve fitting. D) LRP6 occupancy on A549 cells. Occupancy of cell surface LRP6 by indicated antibodies was determined using methods described in Figure S4D. Values represent mean ± SD of a duplicate.

Supplemental Fig. 5. Antagonizing Wnt3a with bsAbs potently inhibits *in vitro* clonogenic activity in a guide antigen-dependent manner.

A) Sphere-cultured HEK293 cells were dissociated into single cells and seeded at a density of 200 cells per well in 24-well low attachment plates. Sphere cells were treated with 10% Wnt3a-CM including each indicated antibody (250 nM) and incubated for 2 weeks. The number of spheres (> 100 μm) was counted from each well (left and middle

panels), and then spheres were dissociated and stained with Calcein-AM (2 μM) for 1 h. Viable cells from spheres were gated on FACS plot and the number was quantified for each experimental group (right panel). B) Tumor-sphere formation assay to determine guide antigen-specific anti-Wnt signaling by bsAbs. Spheres generated from $ALCAM^{High}$ HCT116 or $ALCAM^{Low}$ HT29 colon cancer cell line were dissociated into single cells and seeded as described in (A), and incubated in sphere-forming medium supplemented with 10% Wnt3a-CM with each antibody (250 nM) or XAV939 (50 μM). The number of spheres (> 100 μm) in each well was separately counted (left and middle panels) and representative whole-well images are shown (right panel). *N.S*, not significant. Values in A and B represent the average proportion of counted sphere numbers or viable cell numbers. Error bars denote SD of n = 3. *P<0.05 and **P<0.01.

Supplemental Fig. 6. E34N19/H3 bsIgG effectively inhibits cancer cell migration.

Representative images from wound-healing migration assays. Confluent A549 cells seeded on a 96-well plate were scratched and treated with varying concentrations of indicated antibodies or XAV939 (as a positive control) for 48 h in 20% Wnt3a-CM. Cell images were taken at the indicated time points and wound area was quantified by ImageJ.

Supplemental Fig. 7. H3/E34N19 shows a potent anti-proliferation activity by destabilizing β-catenin.

Guide antigen ALCAM-expressing A549 (A) or HCT116 cells (B) were seeded in a 96-well plate and treated with 10% Wnt3a-CM and indicated concentrations of H3/E34N19 TaFv-Fc or E34N19 scFv-Fc, respectively. Cells were incubated for 4 days and stained

with Calcein-AM. Cell viabilities were measured and normalized against the control group without antibody treatment. C) Inhibition of β-catenin and c-Myc levels by H3/E34N19 treatment. A549 cells were incubated with 10% Wnt3a-CM with and without H3/E34N19. After 24 h incubation, phosphorylated LRP6 (p-LRP6) or active β-catenin and c-Myc levels were analyzed by Western blot.

Supplemental Fig. 8. The guided bispecific does not inhibit Wnt signaling on normal cells and normal stem cells that express a low level of the guide antigen.

A) The anti-LRP6E3E4 antibody E34N19 found to bind to both human and murine LRP6. Recombinant human LRP6E3E4-Fc (hLRP6E3E4), LRP6E1E2-Fc (hLRP6E1E2, as a control) and the mouse full-length LRP6-Fc (mLRP6, R&D systems) were diluted to 5 μg/ml in PBS and coated on a 96 well Maxisorp ELISA plate (Nunc) at 4°C overnight. The plate was blocked by 2% skim milk in PBS at RT for 1h, further incubated with biotinylated E34N19 and an isotype control non-binding antibody (Ctrl Ab), both at 5 μg/ml, at RT for 1h, washed three times with 0.1% PBS-Tween20 and incubated with HRP-conjugated streptavidin (Sigma Aldrich) at RT for 1h. TMB substrate (Thermo Scientific) was used for colorimetric reaction and 1 N HCl (100 μl/well) was added to stop the reaction after 3 min. O.D. values at 450 nm were determined by plate reader Synergy HT (BioTek). B) The anti-guide antibody RYR binds to both human and murine antigen. FACS analysis was performed on murine colon cancer cell line CT26 that express EphA2. Panels (C) and (D) show assessment of guide antigen expression on MC3T3-E1 and C3H/10T1/2, respectively. Binding of RYR (anti-EphA2) and E34N19 (anti-LRP6) (each at 100 nM) was analyzed by FACS. MFI values (after background

subtraction) were used to calculate the guide to effector ratio as indicated under the histogram. Panels (E) and (F) show results of STF reporter assay on MC3T3-E1 and C3H/10T1/2, respectively. Both cell lines were transfected with the STF reporter plasmid, and treated with antibodies at indicated concentrations in Wnt3a-CM. Data in duplicates were analyzed by One-Way ANOVA using the Tukey's method, which showed no significant difference between any of the study groups.

Supplemental Fig. 9. Guided bsAbs are potent in inhibiting Wnt3a/RSPO1-induced Wnt signaling stimulation.

A) STF reporter assay to determine anti-Wnt signaling by H3/E34N19 under a Wnt3a/RSPO1-provided condition. HEK293 cells were transfected with STF reporter and Wnt3a-expression constructs, followed by treatment with recombinant RSPO1 (100 ng/ml) and varying concentrations of the TaFv-Fc. The IC50 value measured for H3/E34N19 was 327 pM. In contrast, the IC50 value for RYR/E34N19 or C10/E34N19 was estimated to be about 50 nM or more than 100 nM, respectively. Values represent mean ± SD for n =3. B) Western blot analysis to determine anti-Wnt signaling activity of bsAbs under the Wnt3a/RSPO1-provided condition. A549 cells were incubated with Wnt3a-CM, recombinant RSPO1 (100 ng/ml), and each indicated antibody (100 nM) for 24 h. Cells were fractionated into a membrane (for p-LRP6) and cytosolic (for active β-catenin and β-actin) fraction. Band intensities of p-LRP6 and β-catenin in each determination were normalized against each β-actin level and described under each band. C) Transwell invasion assay. Representative images from each duplicate, corresponding to Fig. 6D. PC3 cells were seeded in the upper compartment of Transwell chambers and

treated with RYR/E34N19, H3/E34N19, and C10/E34N19 (50 μg/ml) for 24 h, with 20% Wnt3a-CM placed in the lower compartment to induce cell invasion. Invaded cells were stained with crystal violet and microscopic images were taken at 10x magnification.

Supplemental Table 1. Summary of cell surface antigen density measurement.

	Antigen density (Mean ABC ± SD, n≥2)			
	**ICAM1**	**EphA2**	**ALCAM**	**LRP6**
**HEK293-EphA2**	10,186 ± 2,310	5,244,589 ± 432,186	268,022 ± 77,453	8,491 ± 210
**HEK293**	8,005 ± 76	12,026 ± 7,278	285,933 ± 23,044	7,746 ± 2,616
**A549**	7,223 ± 510	30,902 ± 1,774	874,813 ± 40,631	7,341 ± 1,078
**PC3**	117,123 ± 3,982	1,243,814 ± 407,118	891,442 ± 248,550	6,479 ± 1,077
**HCT116**	8,534 ± 780	214,109 ± 19,430	708,341 ± 94,425	6,382 ± 1,787
**HT29**	3,752 ± 72	63,147 ± 863	20,227 ± 779	6,444 ± 335

Supplemental Table 2. Summary of guide antigen density, guide/effector ratio, apparent K_D and anti-Wnt signaling activity (IC50) for the guided bsAb.

Guide antigen	Guided bsAb	Cell model	Guide antigen density (Mean ABC, n≥2)	Guide/ effector ratio	Apparent K_D (pM)	IC50 (pM)
**ICAM-1**	**M10A12/ E34N19**	HEK293-ICAM-1^{High}	484,486	85.9	789	1.13
		HEK293-ICAM-1^{Int}	205,962	34.3	843	3.76
		HEK293-ICAM-1^{Low}	30,702	4.4	871	16.89
		HEK293	10,016	2.0	7,268	1,183
**ALCAM**	**H3/ E34N19**	HEK293	285,933	36.9	28	15.14
		HEK293-ALCAM^{Int}	93,254	12.5	45.2	67.85
		HEK293-ALCAM^{Low}	49,353	6.6	136	141.1
	**PD32/ E34N19**	HEK293	285,933	36.9	578	118.8
**EphA2**	**RYR/ E34N19**	HEK293-EphA2	5,244,589	617.7	787	4.15
		HEK293-EphA2#8	1,922,502	250.6	1,123	8.36
		HEK293-EphA2^{High}	1,434,711	254.3	1,554	6.55
		HEK293-EphA2^{Int}	614,989	102.3	4,734	12.7
		HEK293-EphA2^{Low}	100,309	14.4	18,090	24.45
		HEK293	5,797	0.8	15,960	1,346

Figure S1

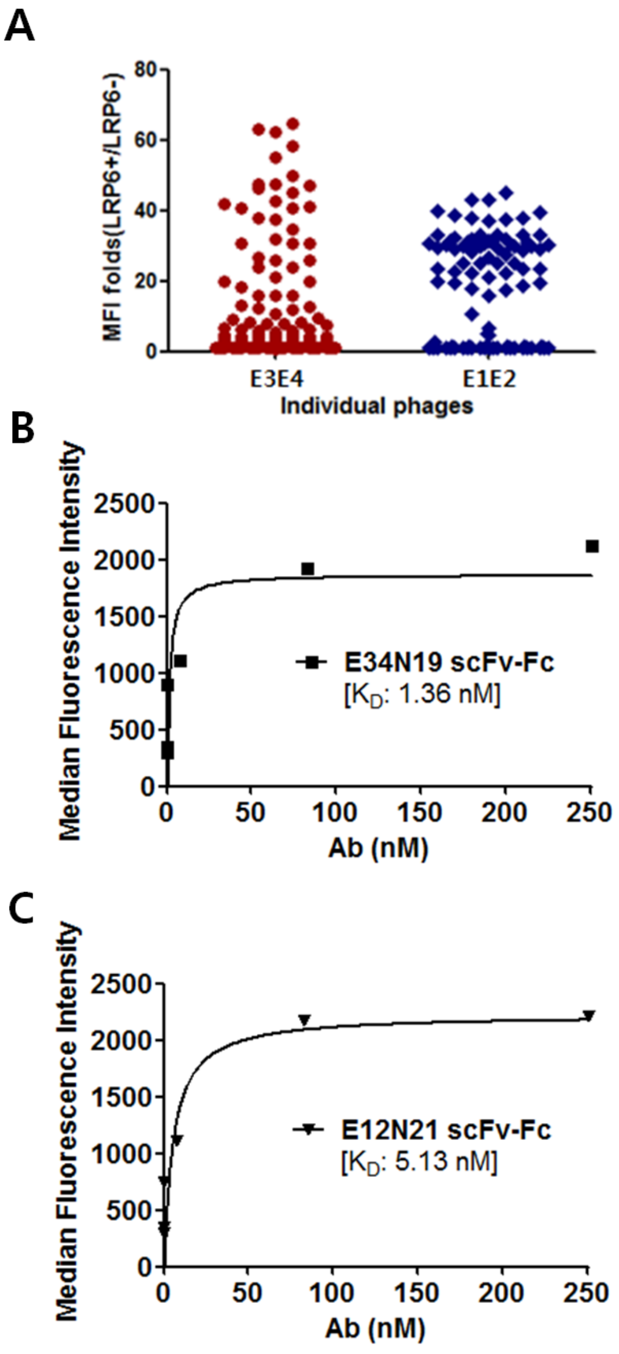
A
MFI folds(LRP6+/LRP6-)
80
60
40
20
0
E3E4
E1E2
Individual phages
B
Median Fluorescence Intensity
2500
2000
1500
1000
500
0
0
50
100
150
200
250
Ab (nM)
E34N19 scFv-Fc
[KD: 1.36 nM]
C
Median Fluorescence Intensity
2500
2000
1500
1000
500
0
0
50
100
150
200
250
Ab (nM)
E12N21 scFv-Fc
[KD: 5.13 nM]

Figure S2

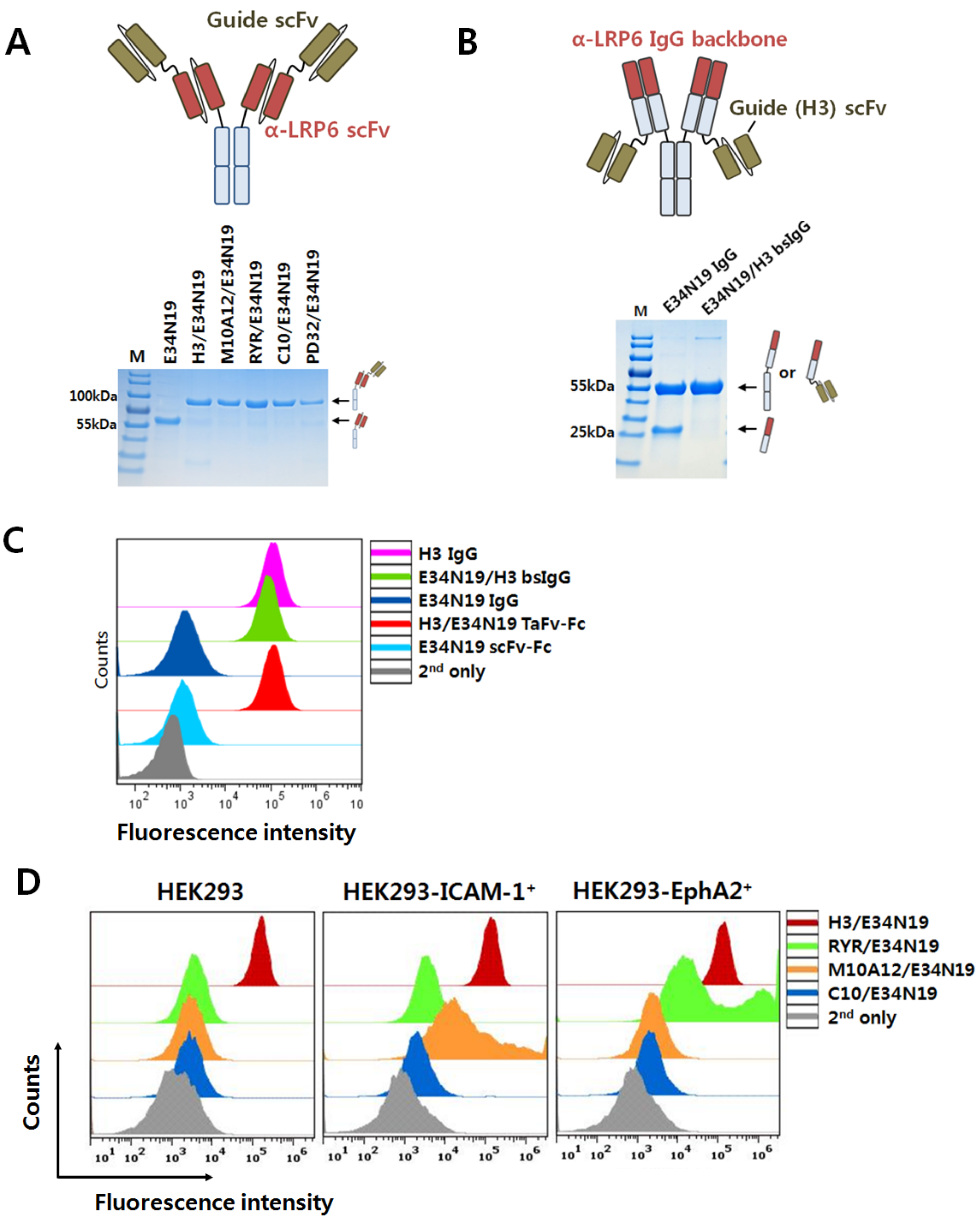
A
Guide scFv
α-LRP6 scFv
M
E34N19
H3/E34N19
M10A12/E34N19
RYR/E34N19
C10/E34N19
PD32/E34N19
100kDa
55kDa
B
α-LRP6 IgG backbone
Guide (H3) scFv
M
E34N19 IgG
E34N19/H3 bsIgG
55kDa
25kDa
or
C
H3 IgG
E34N19/H3 bsIgG
E34N19 IgG
H3/E34N19 TaFv-Fc
E34N19 scFv-Fc
2nd only
Counts
Fluorescence intensity
D
HEK293
HEK293-ICAM-1+
HEK293-EphA2+
H3/E34N19
RYR/E34N19
M10A12/E34N19
C10/E34N19
2nd only
Counts
Fluorescence intensity

Figure S3

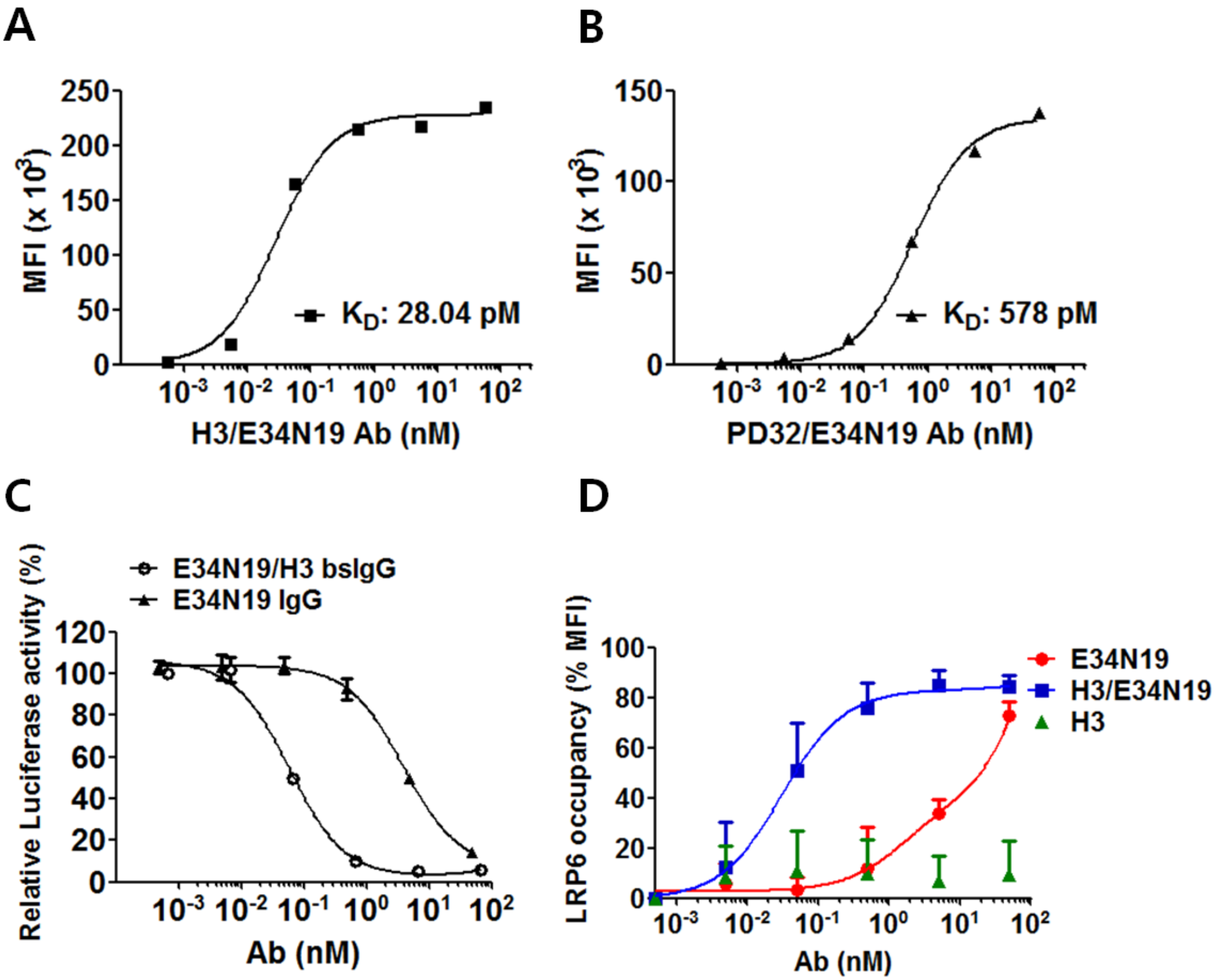

A
250
200
150
100
50
0
MFI (x 10^3)
K_D: 28.04 pM
10^{-3} 10^{-2} 10^{-1} 10^{0} 10^{1} 10^{2}
H3/E34N19 Ab (nM)
B
150
100
50
0
MFI (x 10^3)
K_D: 578 pM
10^{-3} 10^{-2} 10^{-1} 10^{0} 10^{1} 10^{2}
PD32/E34N19 Ab (nM)
C
E34N19/H3 bsIgG
E34N19 IgG
120
100
80
60
40
20
0
Relative Luciferase activity (%)
10^{-3} 10^{-2} 10^{-1} 10^{0} 10^{1} 10^{2}
Ab (nM)
D
E34N19
H3/E34N19
H3
100
80
60
40
20
0
LRP6 occupancy (% MFI)
10^{-3} 10^{-2} 10^{-1} 10^{0} 10^{1} 10^{2}
Ab (nM)

Figure S4

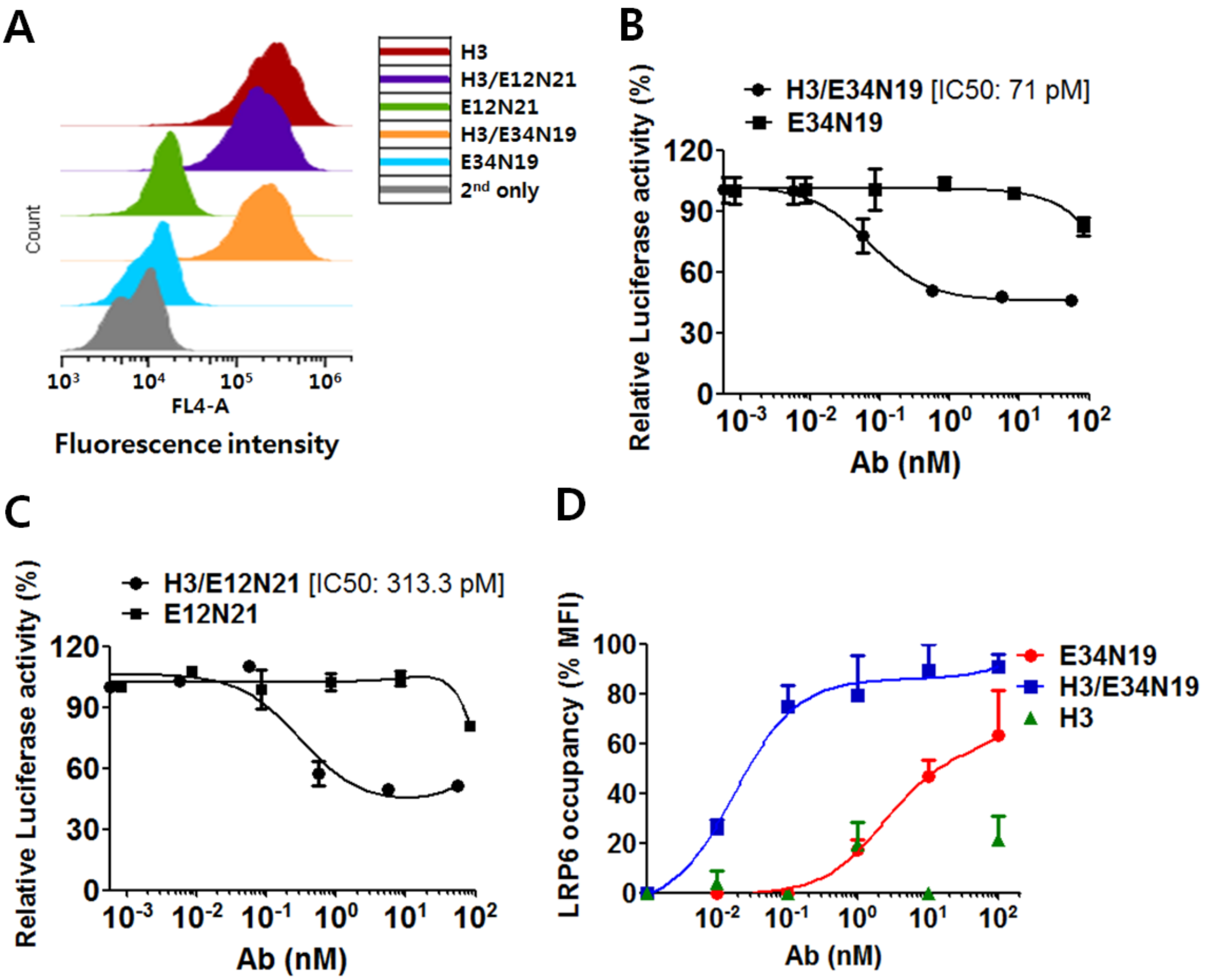
A
H3
H3/E12N21
E12N21
H3/E34N19
E34N19
2nd only
Count
FL4-A
Fluorescence intensity
B
H3/E34N19 [IC50: 71 pM]
E34N19
Relative Luciferase activity (%)
Ab (nM)
C
H3/E12N21 [IC50: 313.3 pM]
E12N21
Relative Luciferase activity (%)
Ab (nM)
D
E34N19
H3/E34N19
H3
LRP6 occupancy (% MFI)
Ab (nM)

Figure S5

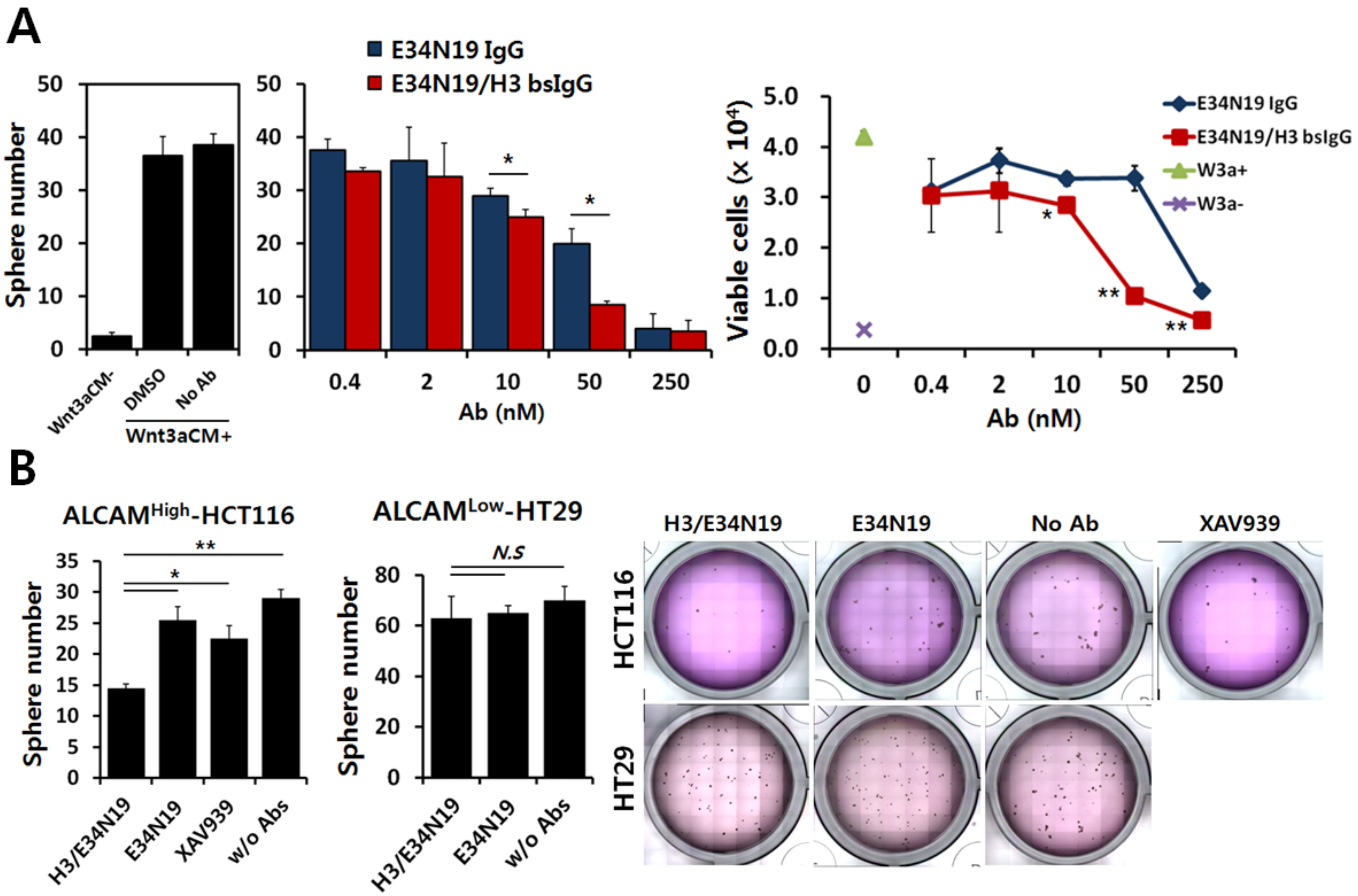
A
Sphere number
Wnt3aCM-
DMSO
No Ab
Wnt3aCM+
E34N19 IgG
E34N19/H3 bsIgG
Ab (nM)
Viable cells (x 10⁴)
W3a+
W3a-
B
ALCAM^High-HCT116
ALCAM^Low-HT29
N.S
H3/E34N19
E34N19
XAV939
w/o Abs
No Ab
HCT116
HT29

Figure S6

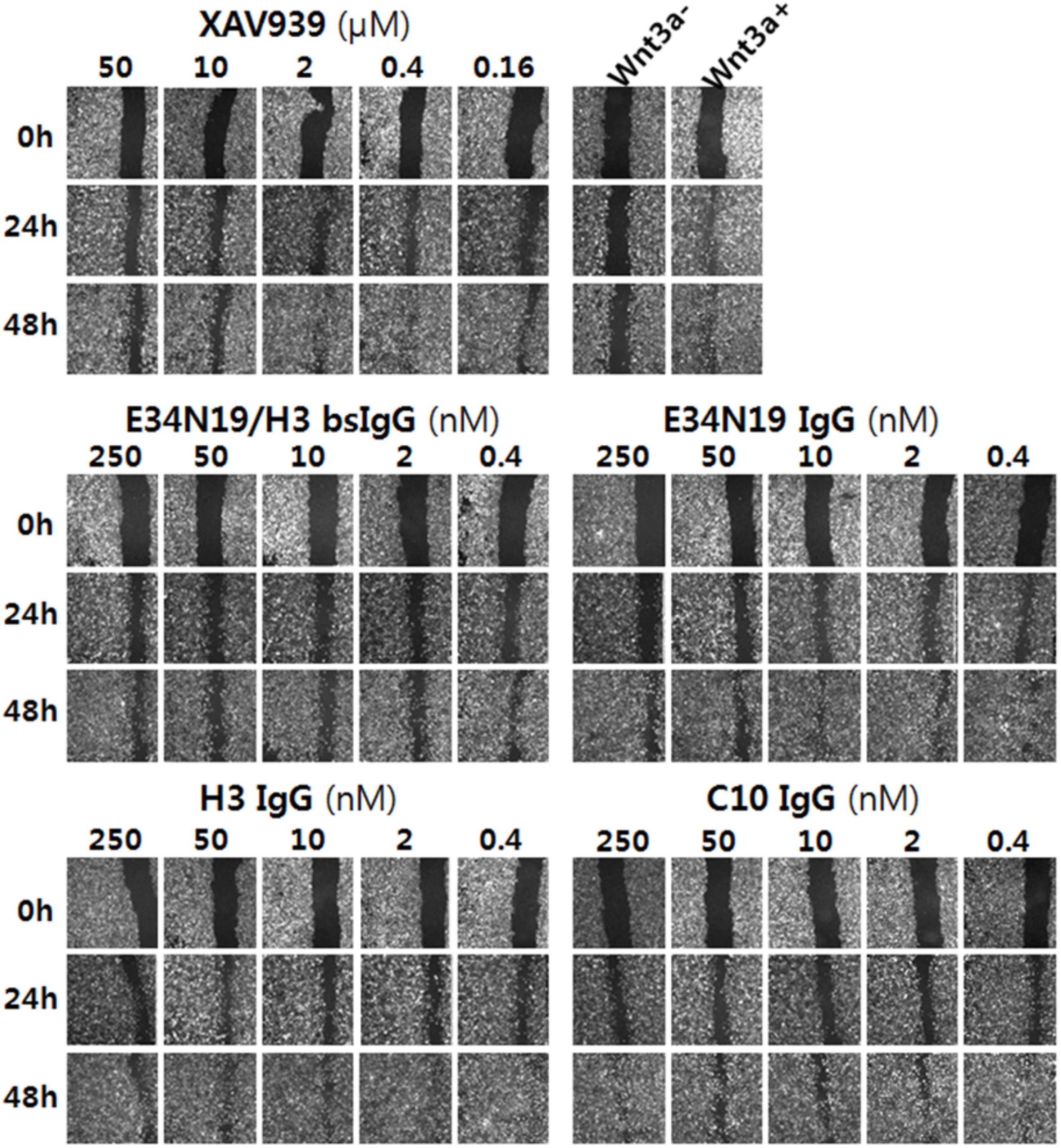
XAV939 (μM)
50
10
2
0.4
0.16
Wnt3a-
Wnt3a+
0h
24h
48h
E34N19/H3 bsIgG (nM)
250
50
10
2
0.4
E34N19 IgG (nM)
250
50
10
2
0.4
0h
24h
48h
H3 IgG (nM)
250
50
10
2
0.4
C10 IgG (nM)
250
50
10
2
0.4
0h
24h
48h

Figure S7

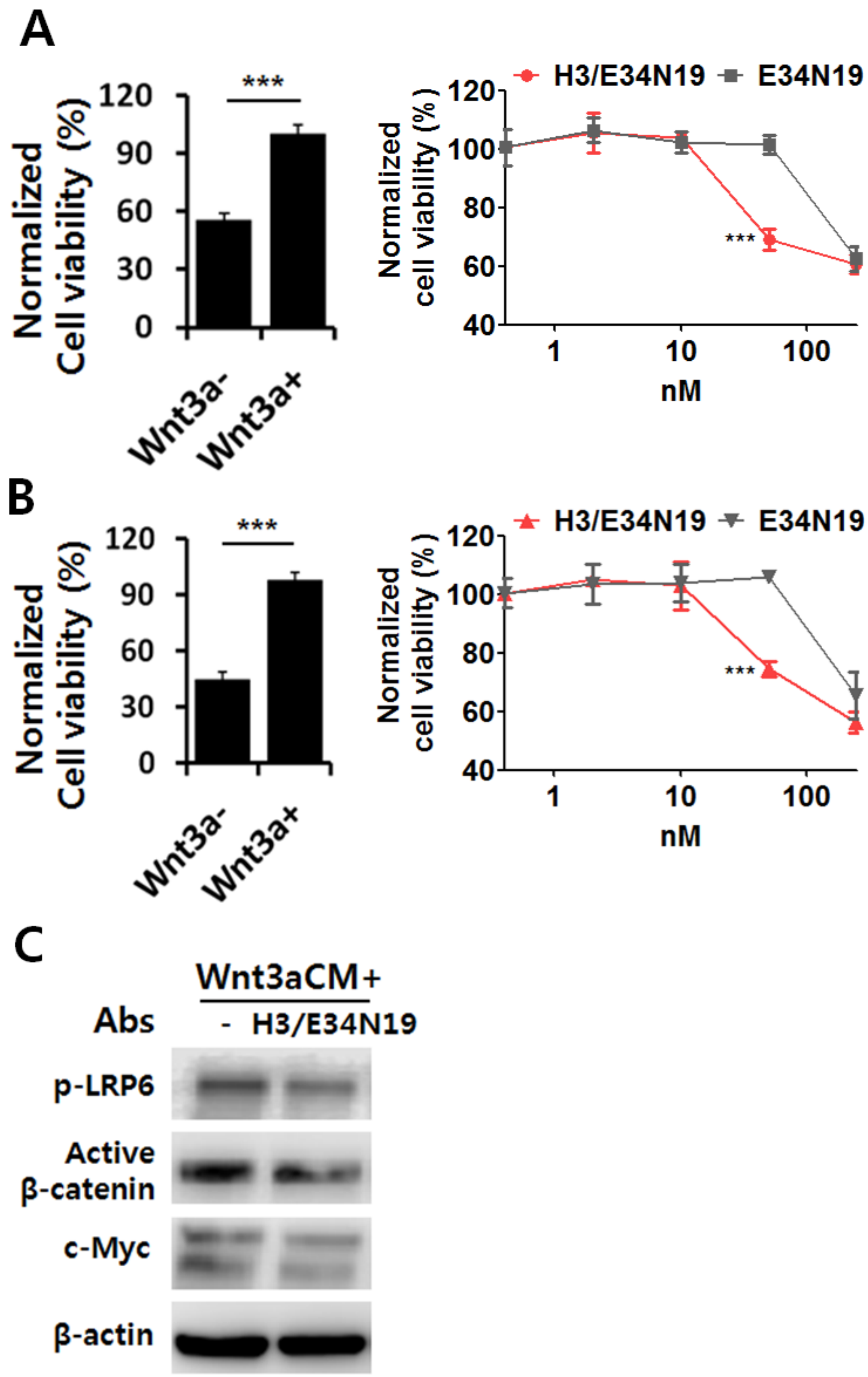
A
Normalized Cell viability (%)
120
90
60
30
0
***
Wnt3a-
Wnt3a+
H3/E34N19
E34N19
Normalized cell viability (%)
120
100
80
60
40
***
1
10
100
nM
B
Normalized Cell viability (%)
120
90
60
30
0
***
Wnt3a-
Wnt3a+
H3/E34N19
E34N19
Normalized cell viability (%)
120
100
80
60
40
***
1
10
100
nM
C
Wnt3aCM+
Abs
-
H3/E34N19
p-LRP6
Active β-catenin
c-Myc
β-actin

Figure S8

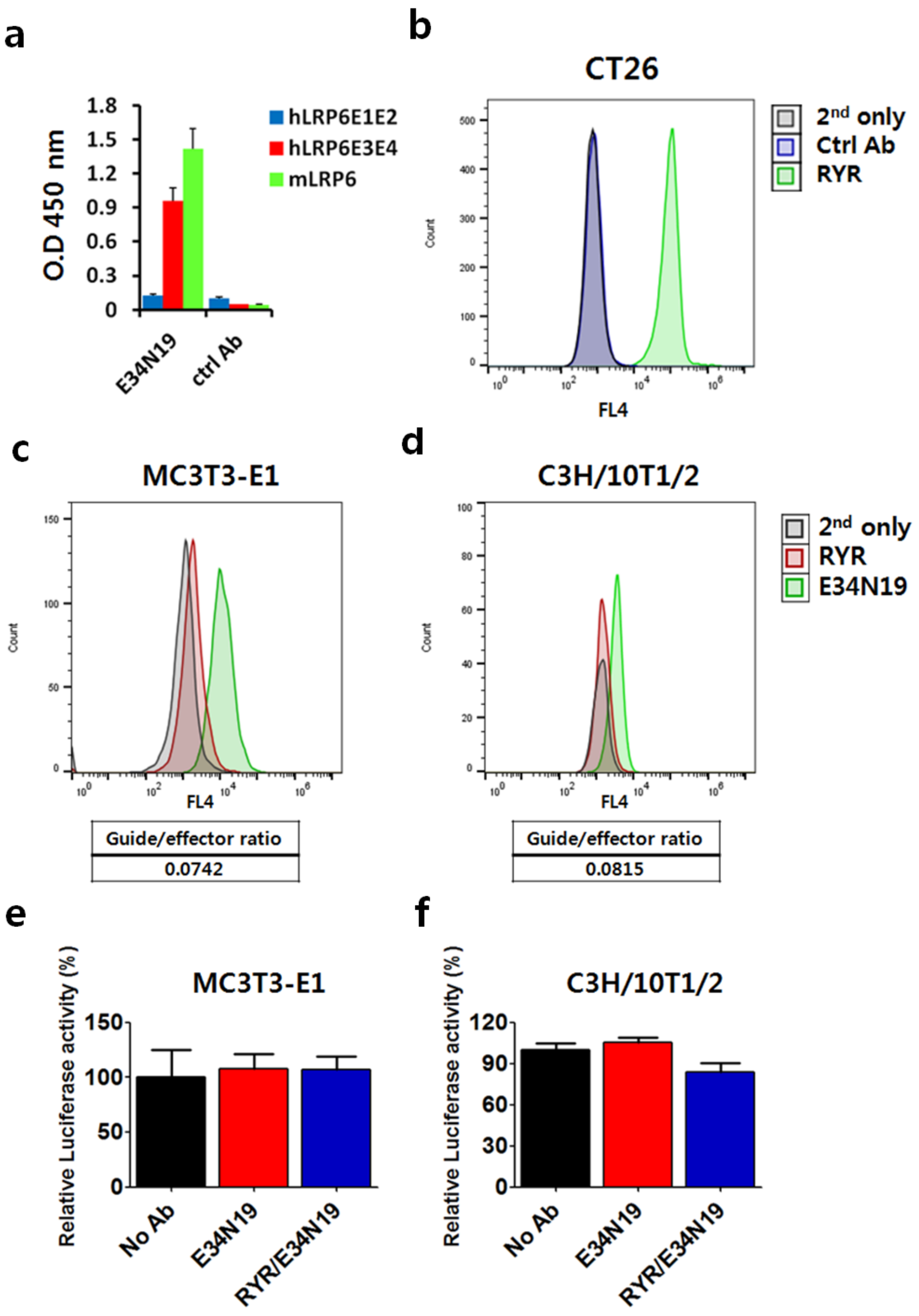
a
O.D 450 nm
1.8
1.5
1.2
0.9
0.6
0.3
0
hLRP6E1E2
hLRP6E3E4
mLRP6
E34N19
ctrl Ab
b
CT26
Count
FL4
2nd only
Ctrl Ab
RYR
c
MC3T3-E1
Count
FL4
Guide/effector ratio
0.0742
d
C3H/10T1/2
Count
FL4
2nd only
RYR
E34N19
Guide/effector ratio
0.0815
e
MC3T3-E1
Relative Luciferase activity (%)
150
100
50
0
No Ab
E34N19
RYR/E34N19
f
C3H/10T1/2
Relative Luciferase activity (%)
120
90
60
30
0
No Ab
E34N19
RYR/E34N19

Figure S9

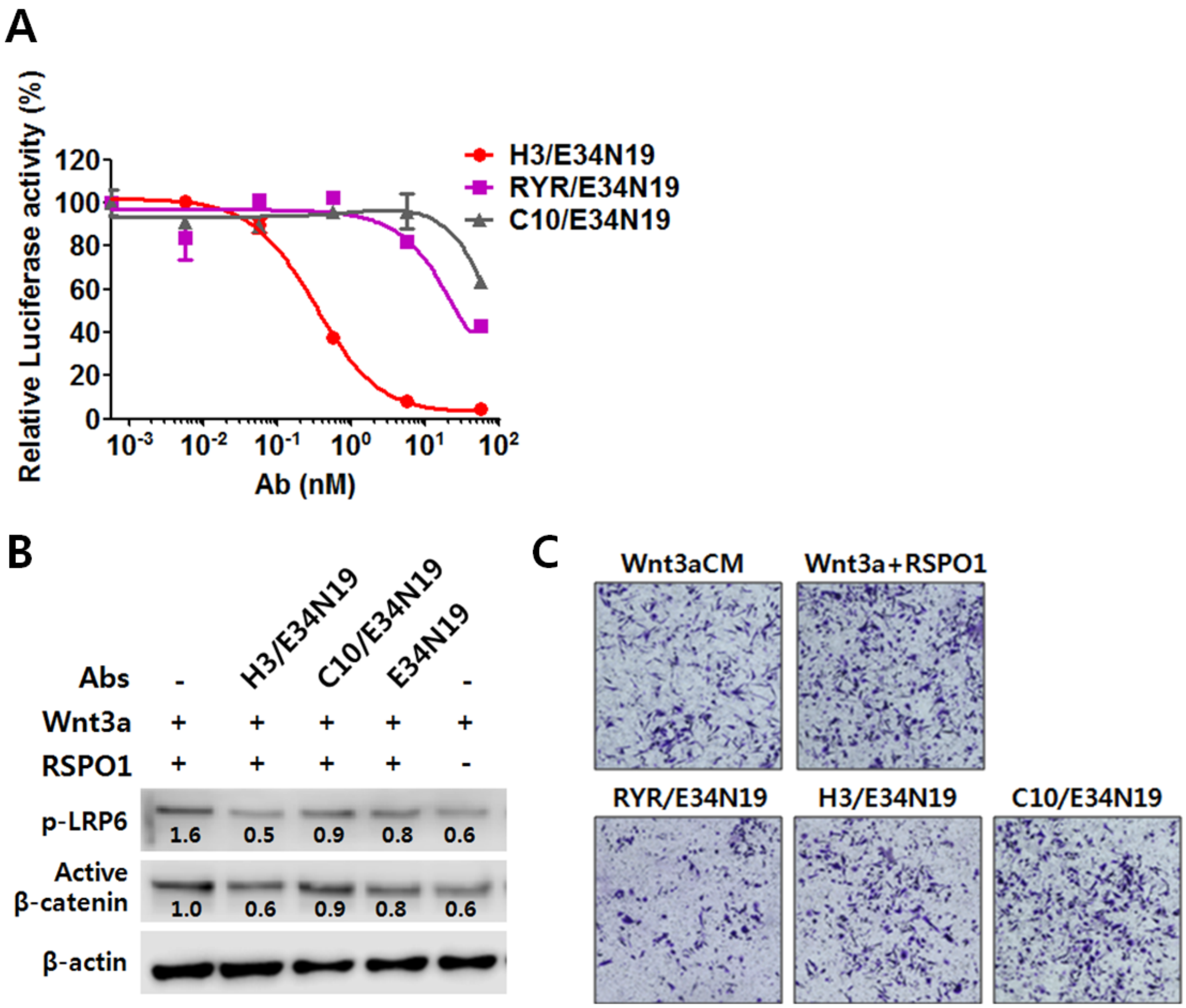
A
Relative Luciferase activity (%)
120
100
80
60
40
20
0
10-3
10-2
10-1
100
101
102
Ab (nM)
H3/E34N19
RYR/E34N19
C10/E34N19
B
Abs
-
H3/E34N19
C10/E34N19
E34N19
-
Wnt3a
+ + + + +
RSPO1
+ + + + -
p-LRP6
1.6 0.5 0.9 0.8 0.6
Active β-catenin
1.0 0.6 0.9 0.8 0.6
β-actin
C
Wnt3aCM
Wnt3a+RSPO1
RYR/E34N19
H3/E34N19
C10/E34N19
